# Blinded, Multicenter Evaluation of Drug-induced Changes in Contractility Using Human-induced Pluripotent Stem Cell-derived Cardiomyocytes

**DOI:** 10.1093/toxsci/kfaa058

**Published:** 2020-05-18

**Authors:** Umber Saleem, Berend J van Meer, Puspita A Katili, Nurul A N Mohd Yusof, Ingra Mannhardt, Ana Krotenberg Garcia, Leon Tertoolen, Tessa de Korte, Maria L H Vlaming, Karen McGlynn, Jessica Nebel, Anthony Bahinski, Kate Harris, Eric Rossman, Xiaoping Xu, Francis L Burton, Godfrey L Smith, Peter Clements, Christine L Mummery, Thomas Eschenhagen, Arne Hansen, Chris Denning

**Affiliations:** k1 Department of Experimental Pharmacology and Toxicology, University Medical Center Hamburg Eppendorf, 20246 Hamburg, and DZHK (German Center for Cardiovascular Research), Partner Site, Hamburg/Kiel/Lübeck, Germany; k2 Department of Anatomy and Embryology, Leiden University Medical Center, 2333 ZD, Leiden, The Netherlands; k3 Department of Stem Cell Biology, University of Nottingham, University Park, Nottingham NG7 2RD, UK; k4 Ncardia, 2333 BD, Leiden, The Netherlands; k5 Clyde Biosciences Ltd, Biocity Scotland, Newhouse, Lanarkshire ML1 5HU, UK; k6 GlaxoSmithKline, Collegeville, Pennsylvania 19426; k7 NC3Rs, London NW1 2BE, UK; k8 Institute of Cardiovascular and Medical Sciences, University of Glasgow, Glasgow G12 8QQ, UK; k9 GlaxoSmithKline, David Jack Centre for R&D, Ware, Hertfordshire SG12 0DP, UK; k10 Department Applied Stem Cell Technologies, University of Twente, 7500 EA Enschede, The Netherlands

**Keywords:** human-induced pluripotent stem cells, cardiomyocytes, contractility, safety pharmacology, inotropy, alternatives to animal testing, inotropy, predictive toxicology, CRACK-IT project, electrophysiology

## Abstract

Animal models are 78% accurate in determining whether drugs will alter contractility of the human heart. To evaluate the suitability of human-induced pluripotent stem cell-derived cardiomyocytes (hiPSC-CMs) for predictive safety pharmacology, we quantified changes in contractility, voltage, and/or Ca^2+^ handling in 2D monolayers or 3D engineered heart tissues (EHTs). Protocols were unified via a drug training set, allowing subsequent blinded multicenter evaluation of drugs with known positive, negative, or neutral inotropic effects. Accuracy ranged from 44% to 85% across the platform-cell configurations, indicating the need to refine test conditions. This was achieved by adopting approaches to reduce signal-to-noise ratio, reduce spontaneous beat rate to ≤ 1 Hz or enable chronic testing, improving accuracy to 85% for monolayers and 93% for EHTs. Contraction amplitude was a good predictor of negative inotropes across all the platform-cell configurations and of positive inotropes in the 3D EHTs. Although contraction- and relaxation-time provided confirmatory readouts forpositive inotropes in 3D EHTs, these parameters typically served as the primary source of predictivity in 2D. The reliance of these “secondary” parameters to inotropy in the 2D systems was not automatically intuitive and may be a quirk of hiPSC-CMs, hence require adaptations in interpreting the data from this model system. Of the platform-cell configurations, responses in EHTs aligned most closely to the free therapeutic plasma concentration. This study adds to the notion that hiPSC-CMs could add value to drug safety evaluation.

On average, 37 new drugs are launched to market each year but cost of development has increased from approximately $14M per drug in the 1960s to approximately $1.5Bn now, inflation adjusted (Catapult, 2018; IFPMA, 2017). Attrition rates remain high, with only approximately 2% of the drugs entering phase 1 clinical trials actually progressing to use in patients. A key concern is cardiovascular toxicity, where acute, chronic, and comorbidity effects account for 17% of the 462 drugs withdrawn from market (Onakpoya *et al.*, 2016) and for 41% of the top 200 prescribed drugs being labeled with adverse drug reaction or black box warnings (Fuentes *et al.*, 2018).

Altered cardiac electrophysiology was implicated in the withdrawal of 13 drugs from market between 1990 and 2006 (Shah, 2006). Such events led to International Conference on Harmonization (ICH) S7B guidelines for proarrhythmic risk detection using simplified *in vitro* assays to measure blockade of the rapid repolarization *I*_Kr_ current, commonly known as hERG (Food and Drug Administration, 2005b). Combined with ICH E14 (Food and Drug Administration, 2005a) guidelines on electrocardiogram monitoring, drug withdrawal due to electrical dysfunction has reduced, with no reported incidences since 2007. These approaches have improved safety, but the relatively poor specificity for predicting human outcomes and overconservativism of the assays has raised concern that promising drug candidates may be abandoned too early due to false positives (Gintant, 2011).

Greater predictivity during the early stages of the drug development pipeline will require certain attributes from the chosen assays. These include being of human origin, suitable longevity for acute and chronic studies, compatible with medium-throughput analysis, reflective of working cardiomyocyte physiology and function, and compliant with 3Rs polices. This reduces the attractiveness or relevance of many existing systems, such as those involving animal models. This is also true for human- and animal-derived primary cardiomyocytes, which rapidly dedifferentiate in culture, lose viability, or become overrun with fibroblasts.

Alternative technologies are now showing potential in cardiovascular safety evaluation, with human-induced pluripotent stem cell-derived cardiomyocytes (hiPSC-CMs) as a key modality (Gintant *et al.*, 2017). This is evidenced by their use in disease modeling, drug discovery and cardiac safety studies, which culminated in the Comprehensive *in vitro* Proarrhythmia Assay (CiPA) and Japanese iPS cardiac safety assessment initiatives (Fermini *et al.*, 2016; Kanda *et al.*, 2018; Sager *et al.*, 2014). Using CiPA as an example, the approach proposed was to identify proarrhythmic risk based on several key modalities including: (1) assessment of several major ion channels in transfected cell lines; (2) *in silico* modeling of the ion channel effects; (3) proarrhythmic assessment in hiPSC-CMs; and (4) clinical assessment of electrocardiograms from phase I human studies. Data that emerged from CiPA show that optical- and multielectrode array-based platforms using commercially sourced hiPSC-CMs enable an 87% accuracy in predicting proarrhythmic liability across geographically diverse testing laboratories (Blinova *et al.*, 2018), with similar studies being reported by others (Bot *et al.*, 2018).

In contrast, there have been relatively few studies that have used hiPSC-CM contractility in predictive safety assessment (Pointon *et al.*, 2017). This is surprising because cardiovascular liability of drugs occurs commonly via altered function of the contractile myocardium. Also, monitoring contractility in hiPSC-CMs is currently being done at low throughput or by using surrogate markers (eg, impedance) and, so far, there has been no detailed cross-site validation study to accurately assess the impact of drugs on hiPSC-CM contractility.

Within this context, a public-private partnership “*InPulse CRACK-IT Challenge*” was established between the pharmaceutical company, GlaxoSmithKline (GSK), and a U.K. funding agency, the National Centre for the Replacement, Refinement & Reduction of Animals in Research (NC3Rs). The aim was to develop medium-throughput technology platform that could measure contractility in hiPSC-CMs as a physiologically relevant functional output for use in preclinical drug safety evaluation. Parallel or simultaneous measures for Ca^2+^ handling and/or voltage, potentially with physiological loading, were requested as part of the challenge as optional parameters to multiplex mechanistically relevant endpoints and thereby inform integrated decision making.

To this end, we established a multinational consortium, comprising 4 academic teams in Germany, Holland, and the United Kingdom, along with 2 biotech companies (Clyde Biosciences; Ncardia), GSK and NC3Rs. We describe the process by which 36 drugs were selected, distributed, and formulated, allowing a training set of 8 drugs to be used to unify standard operating procedures (SOPs). This enabled a multicenter study to be undertaken for blinded evaluation of up to 28 drugs with known positive, negative, or neutral inotropic effects, and led to the creation of an interactive web tool to access datasets for contractility, voltage, and/or Ca^2+^ handling (https://bjvanmeer.shinyapps.io/crackit/, last accessed May 11, 2020). Overall, the different platform-cell combinations had an accuracy of 44%–85% in correctly predicting inotropy. With simple refinement of the test conditions, namely by adopting approaches to reduce signal-to-noise ratio, data variability, reduce spontaneous beat rate to ≤ 1 Hz, and/or enable chronic testing, accuracy could be increased to 85%–93%, comparing favorably with that currently possible with *in vivo* animal models.

## MATERIALS AND METHODS

### 

#### 

##### CellOPTIQ: Adult rabbit ventricular cardiomyocytes

Rabbit hearts were excised via a thoracotomy and submerged in a modified Krebs-Henseleit (KH) solution of the following composition (mmol/l): NaCl (130), KCl (4.5), HEPES (5), NaH_2_PO_4_ (0.4), MgCl_2_ (3.5), and Glucose (10), pH 7.25 at 37°C with NaOH. Hearts were removed and perfused retrogradely at 25 ml⋅min^−1^ (37°C) with a modified KH solution containing 0.75 mmol/l [Ca^2+^] for 5 min, followed by a nominally Ca^2+^-free KH solution with 0.1 mmol/l EGTA for 5 min. Hearts were then perfused with KH solution containing 0.24 mmol/l [Ca^2+^], 1 mg/ml collagenase (type I), and 0.06 mg/ml protease (type XIV). After approximately 4–5 min, enzyme was removed and the left ventricular free wall was then cut into strips in the recirculated enzyme solution containing 1% bovine serum albumin before being mixed to yield a single cell suspension. Cells were maintained in either Ca^2+^-free KH solution or 1 mmol/l Ca^2+^ (via stepwise increments) until use. Intact cardiomyocytes in 1.8 mmol/l modified Ca^2+^ KH solution were loaded with FluoVolt (Thermo Fisher Scientific) at 1:3000 dilution for 10 min. The incubation medium was removed and the cells resuspended in a modified KH solution.

Cardiomyocytes were allowed to settle on a coverslip in a bath (35 mm petri-dish) at 37°C. Cells were field stimulated at a frequency of 2 Hz with 2 ms duration voltage pulses delivered to parallel graphite electrodes (stimulation voltage set to 1.5 times the threshold) for 2 min before sampling FluoVolt and cell video for 10 s. After repeating this for 10–12 cells/dish the drug was added to quiescent cells and left for 30 min before returning to the same myocytes and repeating the stimulus and data capture protocol. A parallel set of vehicle (DMSO) time control experiments were also performed FluoVolt fluorescence (490 nm excitation) was measured at a sampling rate of 10 KHz, whereas the image was recorded on a CCD camera at 100 Hz frame rate using > 700 nm light. Sarcomere length and fractional shortening of sarcomere length was subsequently extracted from the image using a FFT-based algorithm.

##### TTM system

The TTM system was developed and used as described previously (van Meer *et al.*, 2019). Briefly, the all-optical fluorescent system consists of a microscope capable of recording sequential frames while switching exposure wavelengths within 1 ms. Using 3 LEDs at 470, 560, and 656 nm this results in an effective recording speed of 333 Hz per parameter. Baselines measurements (7-s recordings) were made by choosing 3 areas per well and saving the positions to measure the same areas after drug incubation. Data were analyzed with custom software offline and automatically to reduce used bias.

Black glass-bottom 96-well plates (Grenier) were coated with 1:100 Matrigel (Sigma-Aldrich) in DMEM F12 (Sigma-Aldrich). Pluricyte hiPSC-CMs were thawed and plated (40 000/well in 100 µl) according to manufacturer’s instructions in Pluricyte Cardiomyocyte Medium. To help the recovery of the cells 1:100 RevitaCell (Sigma-Aldrich) was added to each well. Empty wells around the plated CM were filled with 200 µl of PBS to minimize evaporation. Cells were refreshed with 200 µl PCM medium at day 1 and day 4 or 5 after plating. All measurements for 1 drug were always performed on days 5 and 6 or days 6 and 7 after plating.

hiPSC-CMs were labeled with ANNINE 6-plus (Sensitive Farbstoffe GbR, Germany; stock concentration: 0.7 mM, dilution 1:833), Rhod 3 (Invitrogen; stock concentration 10 mM, dilution 1:833), and CellMask Deep Red (Thermo Fisher Scientific; stock concentration 5 mg/ml, dilution 1:1000) in 50 ml Pluricyte medium for 20 min at 37°C, refreshed (200 ml Pluricyte medium), and given 10 min to recover at 37°C before recording baseline measurements. Drug incubation took place just after baseline measurements by removing 100 µl medium and adding 100 µl drug solution (30 min at 37°C) prepared according to formulation instructions supplied by GSK.

##### CellOPTIQ: hiPSC-CMs

The hiPSC-CMs used were R-PAT (derived and differentiated in house as described previously) (Mosqueira *et al.*, 2018; Smith *et al.*, 2018), and iCell^2^ and Pluricyte (purchased from Cellular Dynamics International and Ncardia). Manufacturer instructions were followed for the commercial hiPSC lines. For iCell^2^, seeding was at 25 000 cells into each well of 96-well plates (Nunclon Delta Surface; Thermo, 167008) and then maintained for 10 days before use in drug evaluation studies. The same plates were used for R-PAT hiPSC-CMs, which were seeded at 40 000 cells/well and maintained until days 20 and 21 of differentiation before use. For Pluricyte hiPSC-CMs, seeding was at 35 000 cells into each well of 96-well plates (Greiner Bio-One; 655087) and then maintained for 8 days before use. Confluent monolayers of R-PAT and iCell^2^ hiPSC-CMs were changed into serum-free medium (SFM) (Dulbecco’s Modified Eagle Medium [Gibco, 21969035] + 10 mM galactose [Sigma-Aldrich, G0750] and 1 mM sodium pyruvate [Sigma-Aldrich, P2256] 24 h before testing). Pluricyte hiPSC-CMs were changed into 50% SFM and 50% Pluricyte Cardiomyocyte Medium (serum-free) 24 h before day of testing, and on the day of testing were changed into SFM. For the refined conditions described for R-PAT hiPSC-CMs, plating and maintenance were as before but cells were received fresh RPMI-B27 medium 24 h before testing instead of SFM.

hiPSC-CMs in 96-well plate were transiently loaded in 50 µl/well of SFM containing FluoVolt (1:200 part B, 1:2000 part A; Life Tech, F10488) for 20 min at 37°C and 5% CO_2_. After incubation, the medium was replaced with 200 μl/well of SFM. Plates were then incubated at 37°C and 5% CO_2_ for 15 min before recording, which were made using a ×40 (NAO.6) objective at 10 KHz. To apply electric field stimulation, a custom-made 8 channel electrode StimStrip (Clyde Biosciences Ltd) was placed in a row of a 96-well plate and connected externally to a box (DC power supply; Lavota). hiPSC-CMs were paced at a frequency of 1.2–1.7 Hz (or 0.7–1 Hz in the refined conditions) with an amplitude of 8 V and a pulse width of 20 ms. Recordings of 10 s were made for each well (contractility was 100 frames/s, hence 1000 frames). Baselines and drug addition were as described for the TTM system. Electrophysiology data were analyzed using CellOPTIQ proprietary software of Clyde Biosciences and were normalized to a maximum amplitude of 1 and minimum of 0 to standardize height for comparison of traces created in OriginPro (OriginLab version 7.5). Contractility data were analyzed based on pixel displacement using an ImageJ plug-in. This plug-in uses a sum of absolute differences algorithm.

##### Engineered heart tissues

To generate EHTs, in-house hiPSC lines, R-PAT, and ERC18, were differentiated using 2D-monolayer- (R-PAT) or 3D-embryoid body- (ERC18) protocol and fabricated as recently described (Breckwoldt *et al.*, 2017; Mosqueira *et al.*, 2018). In brief, casting molds were generated in 24-well plates with 2% agarose (Invitrogen, 15510-019) and Polytetrafluorethylene (PTFE) spacers (EHT Technologies, C0002). PDMS racks (EHT Technologies, C0001) were placed in the 24-well plates so that pairs of PDMS posts reached into each agarose casting mold. A reconstitution mix was prepared consisting of: 1 × 10^6^ hiPSC-CM; 81.9 µl cardiomyocyte medium; 10 µl Matrigel basement membrane matrix (BD 354234); 5.5 µl 2 × DMEM; 0.1 µl Y-27632 (10 mM; Biorbyt orb60104); 2.5 µl bovine fibrinogen (5 mg/ml; Sigma F4753). Reconstitution mix (97 µl) was mixed with prealiquoted 3 µl thrombin (100 U/ml; Biopur BP11-10-1104) and pipetted into the agarose casting mold. GCAMP6f-lentiviral particles (multiplicity of infection: 0.3, functional titer) were included in the EHT reconstitution mix for Ca^2+^ transient analysis. EHTs started to beat coherently after 10–14 days and were analyzed for contraction and Ca^2+^ transient at ≥ 20 days.

EHTs were transferred from culture medium into preequilibrated (37°C, 40% O_2_, 7% CO_2_), modified Tyrode’s solution (in mM, 120 NaCl; 22.6 NaHCO_3_; 5.4 KCl; 5 glucose; 1 MgCl_2_; 0.4 NaH_2_PO_4_; 0.6–1 CaCl_2_; 0.05 Na_2_EDTA; 25 HEPES) for R-PAT and into DMEM (Gibco 21068028; in mM, 110 NaCl; 44 NaHCO_3_; 5 KCl; 25 glucose; 0.8 MgSO_4_; 0.9 NaH2PO4, 0.6–1 CaCl_2_; 25 HEPES, and vitamins and amino acids) for ERC18 EHTs 40 min before analysis to equilibrate to the testing medium. After equilibration, the cumulative concentration response curve was started with baseline recording in preequilibrated medium with Ca^2+^ concentration of 0.6–1 mM.

For long-term contractility analysis of sunitinib, hiPSC-CM EHTs were equilibrated to regular cell culture medium without serum (DMEM, Biochrom F0415; penicillin/streptomycin 1% [volume/volume], Gibco 15140; aprotinin 5 µM, Sigma-Aldrich A1153; insulin 1.7 µM, Sigma-Aldrich I9278; glutamine 2000 µM, Gibco 25030-081; triiodothyronine 0.77 nM, European Commission-Joint Research Center IRMM-469; hydrocortisone 137 µM: Sigma-Aldrich H0888). Medium change, drug addition and video-optical contractility analysis was performed daily and contraction data was normalized to time control.

Contractility analysis was via automated video-optical contractility analysis was performed in 24-well cell culture plates (Nunc, 122475) (Breckwoldt *et al.*, 2017). Video files of EHT contractions were analyzed by automated EHT figure recognition software (EHT Technologies, A0001). Top and bottom ends of EHT contour were identified and followed during the course of recording. Force was calculated based on shortening during contraction, elastic propensity, and geometry of PDMS posts.

Ca^2+^ transient analysis was combined with contraction in 24-well cell imaging plates (Eppendorf, 030741005). Briefly, set up consisted of an inverted fluorescence microscope (Zeiss) with a custom-made, automated, light tight, CO_2_- and temperature-controlled XY-stage with 24-well plate holder. A camera attached to front port of the microscope was used for contraction analysis (×1.25 objective) and a camera attached to side port of the microscope was used to adjust position of the tissue for fluorescence/Ca^2+^ transient measurement (×10 objective). A small predefined area (0.1 × 0.4 mm) in the center of EHT was used to record Ca^2+^ transients. Mercury lamp, GFP filter set, and photomultiplier tube were used to record change in fluorescence in EHTs expressing GCaMP6f (increase fluorescence during contraction phase). Predefined XYZ coordinates for contraction and Ca^2+^ transient were defined and saved in a bespoke software which controlled microscope settings, XYZ coordinates, and recorded automated-, sequential-contraction and Ca^2+^ transients. Cumulative concentration response curves were performed, similar to contractility analysis, for selective concentrations based on results of contractility analysis.

Data were normalized to a pool of time-matched controls (contractility analysis, ERC18: *n* = 22 EHTs/7.0 experiments; R-PAT: 16 EHTs/5.0 experiments; combined contractility- and Ca^2+^ transient-analysis: 21 EHTs/8.0 experiments) used for all contractility/combined contractility- and Ca^2+^ transient-measurements. Data are plotted as relative change to the mean baseline, normalized to pool time control. Statistical significance was evaluated by GraphPad Software, 1-way ANOVA with Dunnett’s multiple comparisons test versus baseline.

##### Web tool

The web tool was build using the “Shiny” package in R Language and Environment for Statistical Computing. Data were analyzed and visualized using the packages “ggplot2,” “reshape,” “Rfast,” “ggpubr,” “RColorBrewer,” “multcomp,” and “tidyverse.”

## RESULTS

### 

#### Rationale for Selection, Handling, and Distribution of Training and Blinded Drug Sets

Driven by the interests of the pharmaceutical industry, and specifically GSK, the 36 drugs in this study (Supplementary Table 1) were selected on basis of: (1) commercial availability; (2) approximate balance in numbers between positive inotropes (PIs), negative inotropes (NIs), and no effect drugs (NE), with respect to contractility; (3) inclusion of false positives or negatives; (4) availability of functional data on inotropic effect on cardiomyocyte and/or heart function from 1 or more species, including human, spanning use *in vitro*, *ex vivo*, preclinically, and clinically; (5) data on free therapeutic plasma concentrations (FTPCs); (6) solubility in DMSO to allow unified testing at a maximum concentration of 0.1% v/v, which does not cause toxicity in hiPSC-CMs; and (7) broad range of modes of action relevant to contractility and toxicity, including modulation of ion channels and pumps in the cell membrane (*I*_NaV1.5_, *I*_Kr_, *I*_KATP_, *I*_CaL_, *I*_f_, NCX, Na^+^/K^+^-ATPase) or sarcoplasmic reticulum (SR) (RYR, SERCA), β1- and β2-adrenoceptors, Ca^2+^ sensitivity, signaling cascades (phosphodiesterase [PDE], adenylyl cyclase, cyclic AMP), energy production (ATP, mitochondrial stress), myofilament response, and myofilaments as well as less well-known mechanisms (eg, inhibition of tyrosine kinases).

All drugs were purchased, formulated and distributed under contractual material transfer agreement by GSK to ensure that the same lot numbers were used by the testing laboratories. Eight drugs were not blinded and used as a “*training set*” to establish the working parameters and protocols for the technology platforms (isoprenaline, nifedipine, digoxin, BayK8644, EMD-57033, ryanodine, thapsigargin, caffeine). The remaining 28 “*test set*” drugs were encoded and blinded by GSK’s Sample Management Technology department, which dealt with handling and distribution as preweighed lots in coded brown glass vials. Samples were formulated according to prescribed, blinded instructions, and using single-shot vials of DMSO where possible to minimize absorption of water from the atmosphere, hence avoid inadvertent drug dilution. Powders and formulated drugs were stored at −80°C, with the solutions undergoing no more than 2 rounds of freeze-thaw and use within 2 weeks.

#### Refining and Establishing Working Parameters With the Drug “Training Set”

The training set of 8 drugs was used to unify the methods for testing, analysis, and presentation (Supplementary Figs. 1–3). This helped to establish SOPs (Supplementary Table 2), which were used for subsequent blinded evaluation of the “*test set*” drugs. Three technology platforms were used, differing in configuration, format of wells, and approach to calculate contractility. These were the Triple Transient Measurement (TTM), CellOPTIQ (CO), and engineered heart tissue (EHT) platforms.

The TTM platform is bespoke and was the only system in this study that could simultaneously measure contractility, electrophysiology, and Ca^2+^ handling. Interlaced 1000 frame/s movies (ie, sequential 1 ms/channel) were recorded from hiPSC-CMs cultured in 2D monolayers in 96-well plates loaded with appropriate dye (Sala *et al.*, 2018; van Meer *et al.*, 2019). The CO is a proprietary system that was used to measure contractility, via bright field images, and electrophysiology, via voltage-sensitive dyes (Duncan *et al.*, 2017). Here, it was used on hiPSC-CMs cultured in 2D monolayers in 96-well plates and isolated adult rabbit cardiomyocytes. Finally, the EHT system is commercially available, using 3D constructs fabricated from hiPSC-CMs using a fibrin hydrogel between 2 polydimethylsiloxane (PDMS) posts and multiplexed in 24-well plates (Mannhardt *et al.*, 2016). Determining Ca^2+^ handling required viral transgenesis of a genetically encoded calcium indicator (GCAMP6f) during EHT fabrication. Whereas pixel displacement was used as a surrogate of contractility in the 2D systems (TTM and CO), 3D EHTs enable force of contraction to be calculated from the extent of deflection of PDMS posts upon each beat.

Action potential duration (APD_30_, APD_90_) and triangulation (APD_90_–APD_30_) were calculated from voltage waveforms to determine whether electrophysiology was altered, including the appearance of arrhythmias. For Ca^2+^ handling, amplitude, time to peak, and decay time were assessed, whereas similar parameters (contraction amplitude [CA], contraction time [CT], and relaxation time [RT]) were derived for contraction (Supplementary Figure 2). Contractility responses were further subdivided (Supplementary Figure 2), such that positive inotropy reflected an *increase* in CA, positive clinotropy a *decrease* in CT and positive lusitropy a *decrease* in RT. The opposite is true for negative responses.

The training set of 8 drugs was used to test and refine protocols. Representative data are shown for the PIs, digoxin/isoprenaline, and the NI, nifedipine (Supplementary Figure 3). For each parameter, the percentage change in drug-treated samples relative to their respective vehicle control was calculated using the formula ([drug/drug baseline]/[vehicle/vehicle baseline])*100–100. Consistent with the mode of action of these drugs, in all cases there was a trend or significant response for positive inotropy (increased CA) in hiPSC-CMs treated with digoxin/isoprenaline but negative inotropy (decreased CA) for those treated with nifedipine (Supplementary Figure 3). Digoxin tended to increase RT in 2D configuration, whereas isoprenaline decreased of both CT and RT in 3D EHTs (Supplementary Figure 3). These studies allowed unification of SOPs, although physical and technical constraints between the platforms meant that some unavoidable differences remained (Supplementary Table 2).

#### Blinded Evaluation and Assignment of Drug “*Test Set*”

Using the SOPs established above, up to 28 blinded drugs were tested in a rank order that was predefined by GSK (Table 1). Contraction was the parameter common to all platforms because the primary aim of the study was to predict whether drugs were positive, negative, or neutral inotropes. Differentiation of the in-house hiPSC lines, R-PAT, and ERC18, provided sufficient hiPSC-CMs to test all available drugs on the CO and/or EHT platforms. Testing was restricted to 10 drugs in rabbit CMs (as a comparator) and in commercial hiPSC-CMs (Pluricyte and iCell^2^) due to cost, availability and timelines, whereas throughput was a limitation of the TTM system. Overall, 9 drugs were tested in common across all platform-cell combinations, which were later unblinded and identified as: PIs, epinephrine, forskolin, levosimendan, pimobendan; NIs, verapamil, sunitinib; NE, acetylsalicylic acid, atenolol, captopril.

**Table 1.** Effects of Compounds on Cardiomyocyte Contractility *In Vitro*

Compound	Rank	Mode of Action	Platform	Cell	CA	CT	RT	Test Range μM	Conc μM	FPTC μM	Known Effect	Blinded Assignment
Epinephrine	1	Nonselective α1-, β1-, and β2-adrenoceptor agonist	CO	Rabbit	+196	−26	+6 ^0.03 µM^	0.01–1	0.1	0.0002–0.05	PI	PI
			TTM	P-Cyte	+53 ^0.03 µM^	−34 ^1 µM^	−9		0.01			PI
			CO	P-Cyte	+18 ^0.3 µM^	+67 ^1 µM^	−36		0.1			NE
			CO	iCell2	−12 ^0.3 µM^	−43	+39		0.03			PI
			CO	R-PAT	+27 ^0.01 µM^	+16	−18		1			NE
			EHT	R-PAT	+22	−16 ^1 µM^	−22 ^0.3 µM^		0.1			PI
			EHT	ERC18	+8	−6 ^1 µM^	−7		0.01			NE
Forskolin	2	Adenylyl cyclase stimulator	CO	Rabbit	−16	−34 ^0.1 µM^	+45	0.1–10	3	0.012–0.024	PI	PI
			TTM	P-Cyte	+72 ^10 µM^	−37 ^3 µM^	−38		0.3			PI
			CO	P-Cyte	+31 ^1 µM^	+55 ^3 µM^	+47		0.3			NE
			CO	iCell2	−44	+64	+44		10			NI
			CO	R-PAT	−31 ^10 µM^	−33	+20		0.1			NE
			EHT	R-PAT	+15 ^10 µM^	−7	−22 ^3 µM^		0.3			PI
			EHT	ERC18	+18 ^3 µM^	−33	−23		10			PI
Levosimendan	3	Calcium sensitizer PDE3 inhibitor K_ATP_ channel agonist	CO	Rabbit	+16 ^0.1 µM^	−33	−25	0.01–1	0.01	0.026–0.35	PI	NE
			TTM	P-Cyte	+42 ^0.1 µM^	+11 ^0.3 µM^	−12		0.03			NE
			CO	P-Cyte	−54	+54	−40		0.03			NE
			CO	iCell2	+14 ^0.3 µM^	+80	+54		0.1			NE
			CO	R-PAT	+23 ^0.1 µM^	−12	22 ^1 µM^		0.01			NE
			EHT	R-PAT	+6	−7	−14 ^0.3 µM^		0.01			NE
			EHT	ERC18	+13 ^1 µM^	−9 ^0.1 µM^	−4		0.01			NE
Pimobendan	4	Calcium sensitizer PDE3 inhibitor	CO	Rabbit	+132 ^1 µM^	+72	+42	1–100	3	0.005–0.01	PI	NE
			TTM	P-Cyte	+55	−31 ^10 µM^	+73		30			NE
			CO	P-Cyte	−23 ^10 µM^	+69	−19		3			NE
			CO	iCell2	+65	+69	−13 ^100 µM^		1			NE
			CO	R-PAT	+18	+26 ^10 µM^	+29		100			NE
			EHT	R-PAT	+12	−3.5	+12 ^100 µM^		1			NE
			EHT	ERC18	+13 ^30 µM^	−13	+14 ^100 µM^		3			PI
Dobutamine	11	α1-, β1-, and β2-adrenoceptor agonist	CO	R-PAT	+39 ^3 µM^	−16 ^10 µM^	+29	0.1–10	0.3	0.07–1	PI	NE
			EHT	R-PAT	+44 ^3 µM^	−25	−22		10			PI
			EHT	ERC18	+18 ^3 µM^	−37 ^10 µM^	+7		0.3			PI
Milrinone	13	PDE3 inhibitor	CO	R-PAT	−17	+16 ^3 µM^	+15 ^100 µM^	1–100	1	0.1–0.15	PI	NE
			EHT	R-PAT	+18	−11	−9		30			PI
			EHT	ERC18	+7	−18	−6 ^30 µM^		100			PI
Omecamtiv mecarbil	14	Cardiac-specific myosin activator	CO	R-PAT	−47	+117	−34 ^0.01 µM^	0.01–1	1	0.05–4.2	PI	NI
			EHT	R-PAT	+53	+37	−26		0.3			PI
			EHT	ERC18	+22	+38	−6 ^0.3 µM^		1			PI
Terbutaline	21	β2-adrenoceptor agonist	CO	R-PAT	+28	−32 ^3 µM^	−35 ^10 µM^	0.1–10	0.3	1.3	PI	NE
			EHT	R-PAT	+17	−24	−35 ^0.3 µM^		3			PI
			EHT	ERC18	+29 ^10 µM^	−30	−17		1			PI
Verapamil	7	L-type calcium channel blocker, hERG blocker	CO	Rabbit	−82 ^0.03 µM^	+24	+61	0.01–1	0.1	0.05	NI	NI
			TTM	P-Cyte	−79	−68	−11		0.1			NI
			CO	P-Cyte	(5/5)Q	(5/5)Q	(5/5)Q		1			NI
			CO	iCell2	(4/5)Q	+103 ^0.1 µM^	(4/5)Q		1			NI
			CO	R-PAT	−98	−91	−88		1			NI
			EHT	R-PAT	(5/5)Q ^0.1 µM^	−7	−6		0.03			NI
			EHT	ERC18	(3/5)Q ^0.3 µM^	−25 ^0.1 µM^	−17		0.1			NI
Doxorubicin	8	Impairs Ca^2+^ transport mechanisms in SR	TTM	P-Cyte	−47	−46	+51	0.1–10	100	1–2	NI	NE
			CO	P-Cyte	−15	+70	+72		100			NE
			CO	iCell2	+65	−39	+57 ^30 µM^		1			NE
			CO	R-PAT	−27 ^100 µM^	+41 ^30 µM^	−12		1			NE
Sunitinib	9	Multitargeted TK inhibitor	CO	Rabbit	−78	+46	+300	0.1–10	10	0.003	NI	NI
			TTM	P-Cyte	+42	−23 ^10 µM^	+28		0.1			NI
			CO	P-Cyte	(5/5)Q	+163 ^0.1 µM^	(5/5)Q		10			NI
			CO	iCell2	−25 ^10 µM^	−43	+62		1			NI
			CO	R-PAT	−20	+34 ^0.1 µM^	+33		10			NE
			EHT	R-PAT	+11	+6	+4 ^0.1 µM^		0.3			NE
			EHT	ERC18	−8	+6	−8		3			NE
Citalopram	16	SSR inhibitor, hERG & L-type calcium channel blocker	CO	R-PAT	(10/10)Q	(10/10)Q	(10/10)Q	1–100	30	0.05	NI	NI
			EHT	R-PAT	(4/4)Q ^10 µM^	−6 ^3 µM^	+5		1			NI
			EHT	ERC18	(5/5)Q ^10 µM^	+2 ^1 µM^	+7		3			NI
Itraconazole	18	Triazole antifungal, mechanism unclear	CO	R-PAT	(5/5)Q	(5/5)Q	(5/5)Q	0.1–10	10	0.00086	NI	NI
			EHT	R-PAT	−51	+14 ^0.1 µM^	−33		3			NI
			EHT	ERC18	(5/5)Q ^10 µM^	+11	−29 ^3 µM^		1			NI
Sorafenib	19	Multitargeted TK inhibitor	CO	R-PAT	(10/10)Q	(10/10)Q	(10/10)Q	0.1–10	10	0.03	NI	NI
			EHT	R-PAT	−50	+14	+6 ^3 µM^		10			NI
			EHT	ERC18	−56	+6 ^3 µM^	−24		10			NI
Ivabradine	20	*I*_f_ inhibitor	CO	R-PAT	+46	+22	+24 ^0.3 µM^	0.1–10	0.1	0.01–0.1	NI	NE
			EHT	R-PAT	−16	−27	+3 ^0.1 µM^		10			NI
			EHT	ERC18	−56	14	+45		10			NI
Flecainide	22	Sodium channel blocker, hERG blocker	CO	R-PAT	+31	+26 ^10 µM^	+29 ^1 µM^	0.1–10	0.1	0.2–0.4	NI	NE
			EHT	R-PAT	(6/6)Q ^3 µM^	−16	−26 ^1 µM^		0.3			NI
			EHT	ERC18	−74 ^10 µM^	−21	+42		3			NI
Phentolamine	24	Nonselective α-adrenoceptor antagonist	CO	R-PAT	(5/5)Q	(5/5)Q	(5/5)Q	1–100	30	2.25	NI	NI
			EHT	R-PAT	5/5)Q ^10 µM^	−18	−28		3			NI
			EHT	ERC18	(5/5)Q	−30	+53		10			NI
Zimelidine	28	SSR inhibitor	EHT	R-PAT	18 ^1 µM^	−15	−22	1–100	3	0.78	NI	NI
			EHT	ERC18	(5/5)Q ^100 µM^	−25 ^30 µM^	+43		3			NI
Acetylsalicylic acid	5	Cyclooxygenase inhibitor	CO	Rabbit	+26	−21	−13	10–1000	100	0.3–2	NE	NE
			TTM	P-Cyte	+34	−8 ^1000 µM^	+24 ^100 µM^		10			NE
			CO	P-Cyte	−21 ^30 µM^	−35	+20		10			NE
			CO	iCell2	−58 ^30 µM^	−44	+47 ^1000 µM^		10			NE
			CO	R-PAT	+23	−26 ^300 µM^	+17 ^1000 µM^		100			NE
			EHT	R-PAT	−14	+8 ^100 µM^	+15		1000			NE
			EHT	ERC18	+8 ^300 µM^	−8	−15		1000			NE
Atenolol	6	β1 > β2 adrenoceptor antagonist	CO	Rabbit	+16	−13	+968 ^0.3 µM^	0.1–10	1	1	NE	NE
			TTM	P-Cyte	+54	+8	−19 ^3 µM^		1			NE
			CO	P-Cyte	−29	−71 ^10 µM^	+69 ^3 µM^		1			NE
			CO	iCell2	−42 ^0.1 µM^	+58	−52		0.3			NE
			CO	R-PAT	−28 ^3 µM^	+11	+11 ^10 µM^		0.3			NE
			EHT	R-PAT	−25	+7 ^1 µM^	+14		10			NE
			EHT	ERC18	+3	−13	−9 ^3 µM^		10			NE
Captopril	10	ACE inhibitor	CO	Rabbit	−91	+72	+212	1–100	100	1–2	NE	NI
			TTM	P-Cyte	+136	−5	−14 ^10 µM^		1			NE
			CO	P-Cyte	+24 ^30 µM^	+132	−38		100			NE
			CO	iCell2	−27	+15 ^3 µM^	−20 ^10 µM^		1			NE
			CO	R-PAT	+18 ^3 µM^	−26	−16 ^30 µM^		1			NE
			EHT	R-PAT	−18	−7 ^100 µM^	−9 ^10 µM^		3			NE
			EHT	ERC18	+7	−10	−6 ^1 µM^		3			NE
Glibenclamide	12	K_ATP_ channel antagonist	CO	Rabbit	+66 ^1 µM^	−27 ^0.3 µM^	+61	0.1–10	0.1	0.02–0.06	NE	NE
			CO	R-PAT	+36	−26 ^10 µM^	+31		0.1			NE
			EHT	R-PAT	+3	+6	−12		1			NE
			EHT	ERC18	+6	+10	−5 ^3 µM^		10			NE
Enalaprilat	15	ACE inhibitor	CO	R-PAT	+54	+20 ^10 µM^	+34	1–100	100	0.12	NE	NE
			EHT	R-PAT	+33	+5 ^1 µM^	−14		30			PI
			EHT	ERC18	+2 ^10 µM^	−3	−4		100			NE
Clonidine	17	α2-adrenoceptor agonist	CO	R-PAT	−24	+26	−27	0.01–1	0.3	0.002–0.004	NE	NE
			EHT	R-PAT	+10	+11 ^0.1 µM^	−12		0.3			NE
			EHT	ERC18	+5 ^0.3 µM^	+5	+4		0.1			NE
Paracetamol	23	Prostaglandin synthesis inhibitor	CO	R-PAT	+19 ^100 µM^	+26	+13 ^1000 µM^	10–1000	30	50	NE	NE
			EHT	R-PAT	−19	−20	−9 ^100 µM^		1000			NE
			EHT	ERC18	+3 ^100 µM^	−9	+10		1000			NE
Tolbutamide	25	K_ATP_ channel antagonist, adenylyl cyclase stimulator	CO	R-PAT	+35	−16 ^10 µM^	+45	1–100	100	20–30	NE	NE
			EHT	R-PAT	−12	+9	−8 ^10 µM^		1			NE
			EHT	ERC18	+21	−3	−9		100			PI
Pravastatin	26	HMG CoA reductase inhibitor	CO	R-PAT	+53	+42	+12 ^1 µM^	1–100	3	0.018	NE	NE
			EHT	R-PAT	−19	−15 ^30 µM^	13 ^100 µM^		3			NE
			EHT	ERC18	+19	+3 ^30 µM^	−11 ^100 µM^		10			PI
Sildenafil	27	PDE5 inhibitor	CO	R-PAT	+56	+39	+30 ^3 µM^	0.3 − 30	10	0.02	NE	NE
			EHT	R-PAT	−10	−16	+13 ^3 µM^		10			NE
			EHT	ERC18	+9	−4 ^10 µM^	−6		1			NE

Drugs were ranked by GSK personnel to provide a testing order, based on modes of action that were of interest to them. For each platform-cell combination, data listed represent mean maximum measurable percentage change relative to baseline for CA, CT, and RT. The concentration at which this effect occurred is listed but when it was different for CA, CT, and RT information is superscripted. Green indicates the predicted effect matched the known effect on inotropy. Analysis platforms were CO, CellOPTIQEHT, and engineered heart tissueTTM, Triple Transient Measurement. Cardiomyocyte types were rabbit adult cardiomyocytes (as comparator) or hiPSC lines: P-Cyte, Pluricyte (Ncardia); iCell^2^ (Cellular Dynamics International); R-PAT (University of Nottingham); ERC18 (University of Hamburg).

Abbreviations: FPTC, free plasma therapeutic concentration; ACE, angiotensin converting enzyme; PDE, phosphodiesterase; SSR inhibitor, selective serotonin reuptake inhibitor; PI, positive inotrope; NI, negative inotrope; NE, no effect.

**Figure 1.**
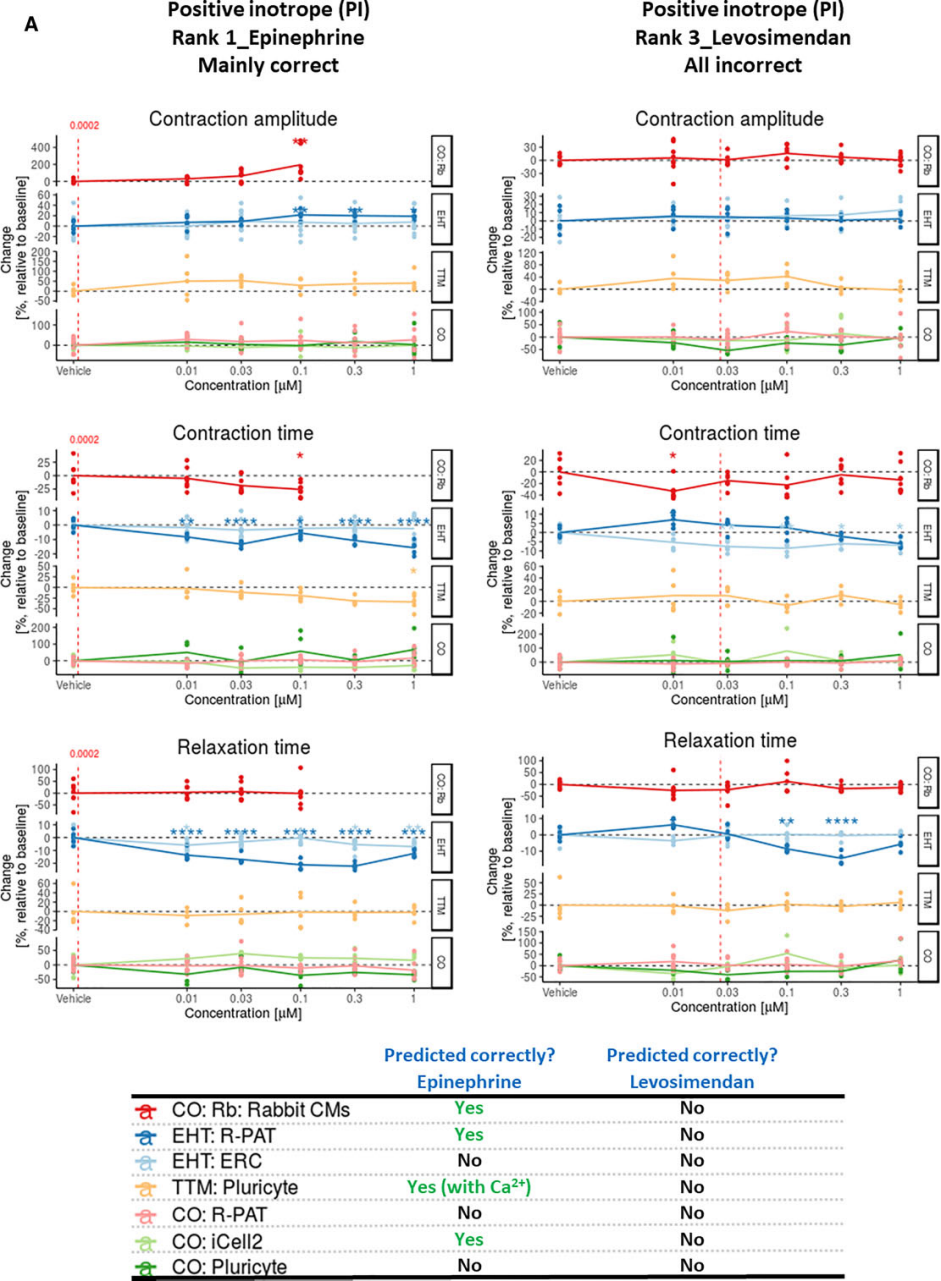
A, Contraction analysis across the platform-cell combinations for PIs. Examples are given to illustrate when some or all platform-cell combinations enable correct or incorrect predictions of the PIs, epinephrine, and levosimendan, respectively. The table indicates where decision making was guided by data from Ca^2+^ transients and/or voltage, with example data provided for epinephrine and verapamil in Supplementary Figures 4A and 4B. Red dotted line is free therapeutic plasma concentration (FTPC). Dunnett’s stats versus vehicle control: *p < .05; **p < .01; ***p < .001; ****p < .0001. B, Contraction analysis across the platform-cell combinations for NIs. Examples are given to illustrate when some or all platform-cell combinations enable correct or incorrect predictions of the NIs, verapamil, and sunitinib, respectively. The table indicates where decision making was guided by data from Ca^2+^ transients and/or voltage, with example data provided for epinephrine and verapamil in Supplementary Figures 4A and 4B. Red dotted line is FTPC. Dunnett’s versus vehicle control: *p < .05; **p < .01; ***p < .001; ****p < .0001. C, Contraction analysis across the platform-cell combinations for NEs. Examples are given to illustrate when some or all platform-cell combinations enable correct or incorrect predictions of the NEs, acetylsalicylic acid, and captopril, respectively. Red dotted line is FTPC. Dunnett’s stats versus vehicle control: *p < .05; **p < .01; ***p < .001; ****p < .0001. Abbreviations: CO, CellOPTIQ; EHT, engineered heart tissue; TTM, Triple Transient Measurement.

All data for contraction, voltage, and Ca^2+^ handling across the various platform-cell combinations are accessible via an interactive web tool via https://bjvanmeer.shinyapps.io/crackit/. Representative data for contractility illustrates the output format for 6 drugs where the response was generally predicted correctly or incorrectly, respectively. Thus, data are shown for the PIs, epinephrine, and levosimendan (Figure 1A), NIs, verapamil, and sunitinib (Figure 1B), and NE drugs, acetylsalicylic acid, and captopril (Figure 1C). In some instances, data from Ca^2+^ and, to a lesser extent, voltage analyses were used to assist in prediction of inotropic response, as was the case for the PI, epinephrine, and the NI, verapamil (Supplementary Figs. 4A and 4B).

Prediction of inotropy involved a 2-day face-to-face meeting between all investigators, with colleagues from GSK overseeing the process as observers rather than contributors. Each platform-cell-drug combination was evaluated individually and a consensus between the research team on the outcome was established using the terminology defined in Supplementary Figure 2 (ie, positive or negative inotropy [CA], clinotropy [CT], and lusitropy [RT]; altered electrophysiology ± arrhythmias [AE ± *]; altered Ca^2+^). In some cases, decisions were based on trends rather than statistical significance. For example, this was true for TTM: Pluricyte evaluation of epinephrine (Figure 1A, see CA and CT). Once predictions were finalized, the document was “locked” and drugs were unblinded by GSK to allow comparison with their known effects (Tables 1 and 2).

**Table 2.** Predictivity Tables and Scores

Platform:Cell Combination		Config.	Nine Drugs in Common				All Drugs Assessed				
			PI (%)	NI (%)	NE (%)	Total (%)	PI (%)	NI (%)	NE (%)	Total (%)	
CellOPTIQ	Rabbit CMs	2D	2/4 (50)	2/2 (100)	2/3 (67)	6/9 (67)	2/4 (50)	2/2 (100)	3/4 (75)	7/10 (70)	Color key of predictivity scores
TTM	Pluricyte hiPSC-CMs		2/4 (50)	2/2 (100)	3/3 (100)	7/9 (78)	2/4 (50)	2/3 (67)	3/3 (100)	7/10 (70)	Predictivity of > 75%
CellOPTIQ	Pluricyte hiPSC-CMs		0/4 (0)	2/2 (100)	3/3 (100)	5/9 (56)	0/4 (0)	2/3 (67)	3/3 (100)	5/10 (50)	
	iCell^2^ hiPSC-CMs		1/4 (25)	2/2 (100)	3/3 (100)	6/9 (67)	1/4 (25)	2/3 (67)	3/3 (100)	6/10 (60)	Predictivity of 50%–74%
	R-PAT hiPSC-CMs		0/4 (0)	1/2 (50)	3/3 (100)	4/9 (44)	0/8 (0)	5/9 (56)	10/10 (100)	15/27 (56)	
EHT	R-PAT hiPSC-CMs	3D	2/4 (50)	1/2 (50)	3/3 (100)	6/9 (67)	6/8 (75)	8/9 (89)	9/10 (90)	23/27 (85)	Predictivity of < 50%
	ERC hiPSC-CMs		2/4 (50)	1/2 (50)	3/3 (100)	6/9 (67)	6/8 (75)	8/9 (89)	8/10 (80)	22/27 (81)	

Collective data are presented for cross comparison between platform-cell combinations relating to the 9 drugs tested in common or all drugs evaluated. Green shading shows predictivity of > 75%, orange of 50%–74%, and red of < 50%. Predictivity is gauged as whether the assignment made by the investigators matched the assignment made by the commercial sponsor, GlaxoSmithKline, and the available literature described in Supplementary Table 1.

Across the 9 drugs evaluated in common, all platforms mainly predicted NE drugs and NIs (Table 2). One exception was sunitinib, which was poorly detected by the EHT platform. This may be due to slower penetration, lower sensitivity, and greater cell community effect of 3D tissues protecting against short-term (30 min) exposure to this chronic toxicant. Poorest predictivity was for PIs, which ranged from 0% to 50% accuracy. Overall predictivity for the 9 drugs ranged from 44% to 78%. This range was similar across all drugs tested (50%–85%), with NE and NI being predicted most accurately (up to 100%; Table 2). The trend was for the EHT:hiPSC-CM combination to be more predictive of PIs (75%) than CO: hiPSC-CM (0%–25%). Together, these data implied that continuous measurements from 3D preparations of hiPSC-CMs yielded more predictive contractility data than cell motion sampled from a 2D culture.

We looked for trends in the data that might point toward ways to improve the process of predicting response. The drug concentration at which the maximum responses were recorded were broadly similar across the TTM, CO, and EHT platforms, irrespective of whether prediction was correct (Table 1). However, we noted that the response range was wider in 2D relative to 3D platforms. Whereas mean maximum measurable percentage changes in CA for the TTM and CO platforms ranged from −100% to +196%, it was −100 to +53% for the EHT platform (Table 1). Where there was an increase in CA, the standard deviation of these mean values was 41.5 versus 12.1 for the 2D versus 3D platforms. A similar pattern was observed for NIs, and for changes in CT and RT. This was corroborated by the wider distribution of data points in the 2D platforms relative to the 3D platform (Figs. 1A–C, Supplementary Figs. 4A and 4B, web tool). This likely relates in part to the cumulative concentration response protocol used for EHTs (Supplementary Table 2).

**Figure 2.**
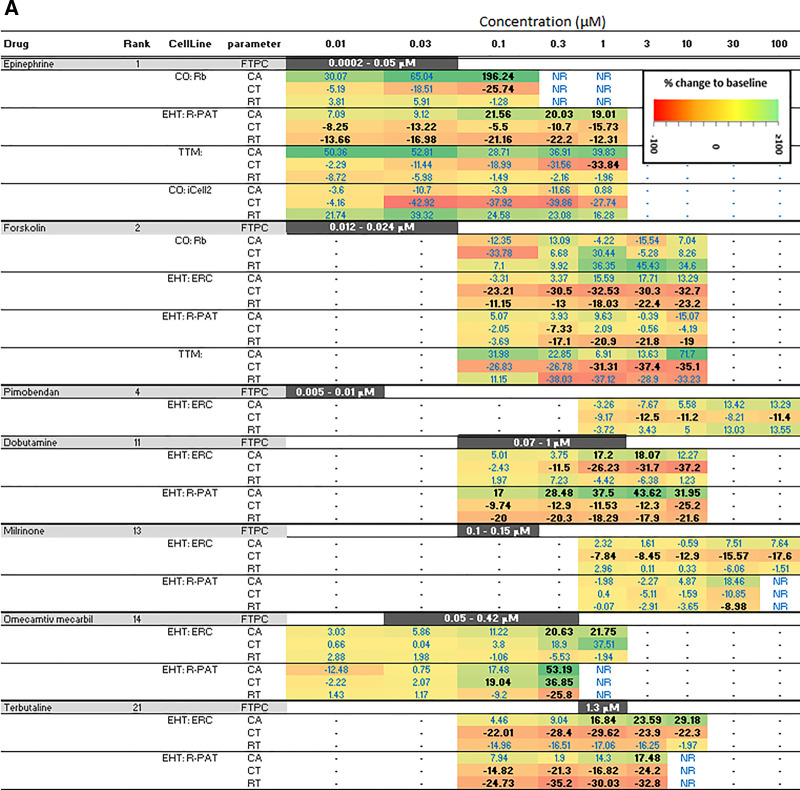
A, Sensitivity of platform-cell combinations for predicting positive inotrope (PIs). Where PIs were correctly predicted, data are presented as percent change relative to baseline for any of the 3 contractility parameters (CA, contraction amplitude; CT, contraction time; RT, relaxation time). The bolded black text indicates where significance was reached using Dunnett’s stats versus vehicle control and *p* < .05. B, Sensitivity of platforms-cell combinations for predicting negative inotropes (NIs). Where NIs were correctly predicted, data are presented as percent change relative to baseline for any of the 3 contractility parameters (CA, contraction amplitude; CT, contraction time; RT, relaxation time). The bolded black text indicates where significance was reached using Dunnett’s stats versus vehicle control and *p* < .05. Abbreviations: CO, CellOPTIQ; EHT, engineered heart tissue; FPTC, free plasma therapeutic concentration; NR, not recorded; TTM, Triple Transient Measurement.

These observations prompted us to examine sensitivity of the different platforms. Where platform-cell combinations correctly predicted inotropy, data were converted into a heat map to reflect the percentage change for PIs (Figure 2A) and NIs (Figure 2B). In 6/7 correctly predicted cardio-active PIs, 1 or more contractile parameters (CA, CT, and/or RT) recorded from EHTs reached statistical significance at concentrations within, or close to, the FTPC range, whereas 2D platforms appeared to be less sensitive (Figure 2A). This is illustrated by epinephrine, where significant changes (*p* of ≤ .05, Dunnett’s post-test) in CT and RT were detected within the FTPC range at a sensitivity of 10- to 100-fold greater than the 2D platforms, and a similar pattern was seen for forskolin (Figure 2A). These trends did not extend to NIs, presumably because on- or off-target cardiac toxicity associated with a proportion of compounds in this group will be occurring after longer incubation times and at higher concentrations than those used for therapeutic benefit, which is reflected in the FTPC.

**Figure 3.**
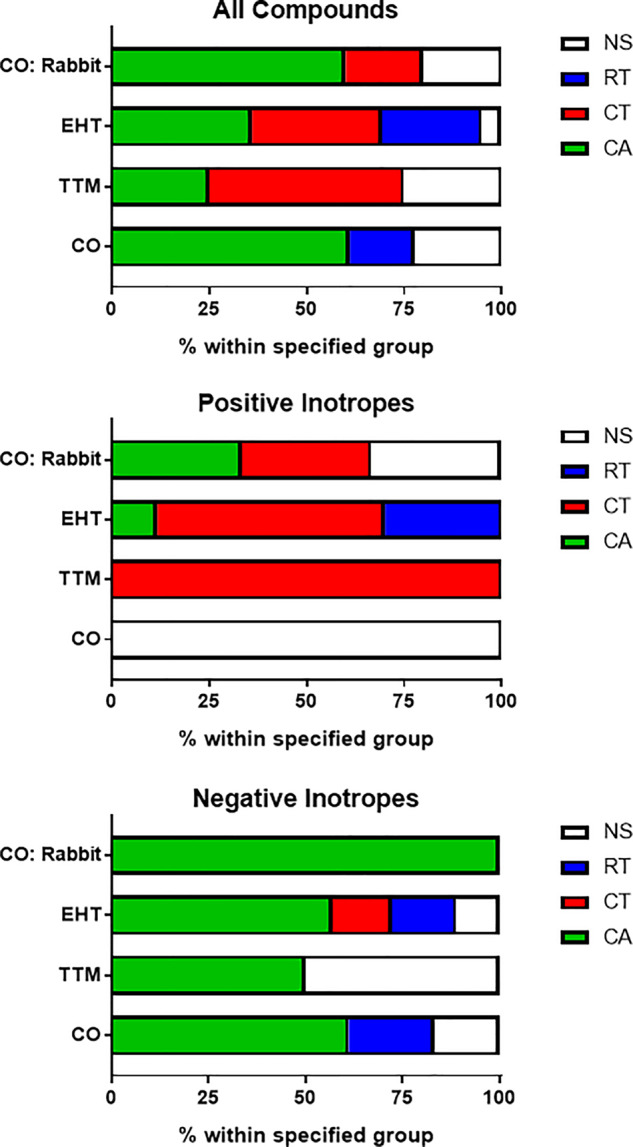
Most informative contractility marker. Parameters that led to the correct prediction of drug responses were assessed. Data show the breakdown by percentage for the parameters (CA, contraction amplitude; CT, contraction time; RT, relaxation time) that reached significance at the lowest concentration (Dunnett’s stats vs vehicle control and *p* < .05). White boxes (NS, not significant) indicate where predictions were made due to a trend rather than reaching significance, and/or by guidance from Ca^2+^ and/or voltage data. Abbreviations: CO, CellOPTIQ; EHT, engineered heart tissue; TTM, Triple Transient Measurement.

Finally, we investigated which parameter was most informative (Figure 3). Where prediction was correct, we asked which parameter within these *in vitro* models reached significance at the lowest drug concentration. Pooled data from both PIs and NIs showed no clear distinction between CA, CT, and RT. However, segregation showed that CA was clearly most informative parameter for NIs. In contrast, CT was highly informative for PIs and particularly those with cAMP-mediated modes of action, with some contribution being derived from RT in hiPSC-CMs and CA in rabbit CMs.

#### Understanding Why Incorrect Assignments Were Made and Improving Test Platforms

The data above highlighted several deficiencies, including: (1) the discrimination of changes in contractility assessed from intermittent video measurements using a motion algorithm on 2D systems was lower than the continuous tension measurements on 3D systems, (2) detection of PIs was challenging, particularly to 2D platforms, and (3) chronic toxicants, such as doxorubicin, were poorly predicted after the acute (30 min) treatment duration.

**Figure 4.**
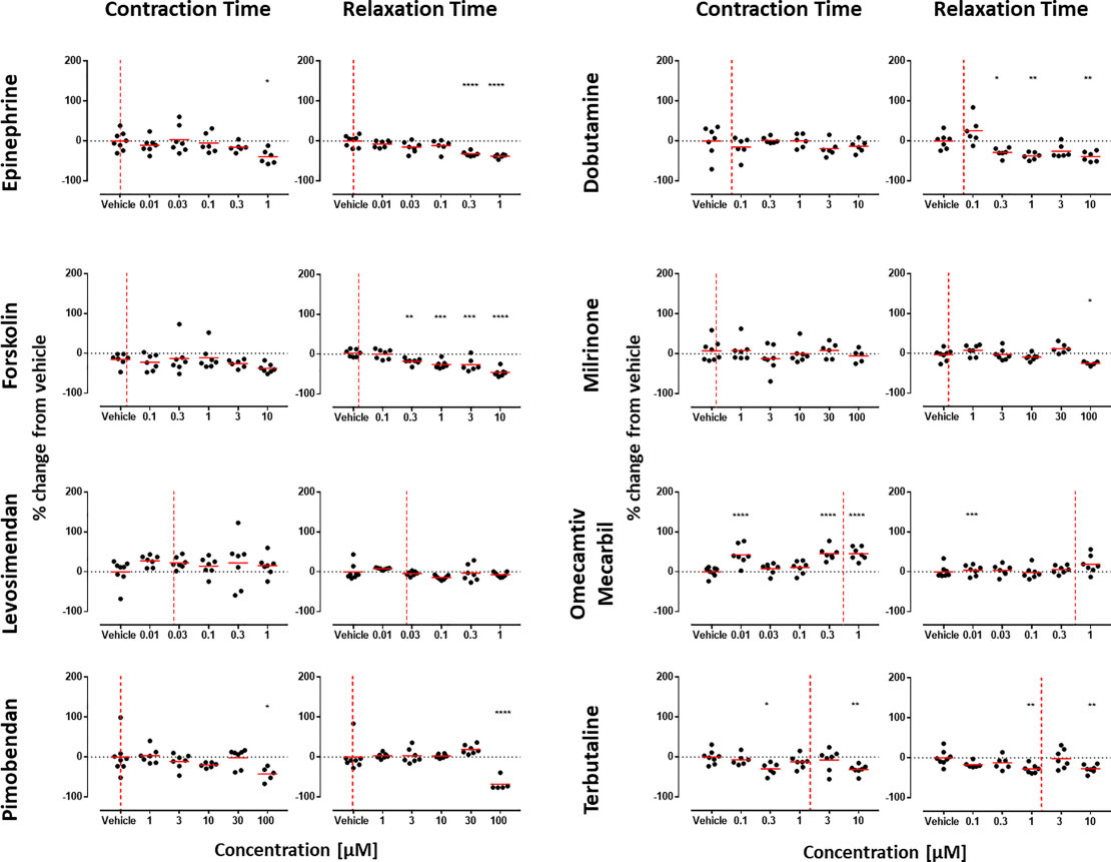
Refined culture conditions increase predictivity of positive inotropes (PIs) in 2D monolayers of hiPSC-CMs. Contraction analysis was carried out on the 8 PIs from the drug test set using the CO:R-PAT platform-cell combination. Only CT and RT are shown because data in Figure 3 showed these to be the most informative parameters for PIs. Whereas testing in serum-/protein-free medium failed to identify any PIs correctly (A, web tool), the slowed beat rate and improved signal-to-noise ratio afforded by culture in RPMI-B27 allowed correct identification of 6/8 PIs by significant decreases in CT and/or RT. Red dotted line is free therapeutic plasma concentration. Dunnett’s stats versus vehicle control: *p < .05; **p < .01; ***p < .001; ****p < .0001.

We reasoned that medium composition may be a contributing factor in signal-to-noise ratio and poor detection of PIs. This was because, on the CO platform, switching from protein-containing (RPMI-B27) to serum-/protein-free medium increased the mean beat rate from 0.7 to ≥ 1.2 Hz (Supplementary Figure 5; *p* ≤ .0001, Mann-Whitney). This was concurrent with increased spread of the data for CA, CT, and RT, wherein the standard deviations for RPMI-B27 versus serum-/protein-free medium were 12.1 versus 32.8, 14.5 versus 20.1, and 10.7 versus 26.9, respectively (Supplementary Figure 5). It has previously been reported in native heart muscle preparations that lower beating rate is associated with a positive force-frequency relationship (up to approximately 2 Hz) and stronger inotropic effects of most PIs (Butler *et al.*, 2015). Therefore, we retested PIs from the test set of drugs using CO:R-PAT, because this platform:cell combination proved poorly predictive (0/8) in the blinded study. In these revised testing conditions, 6/8 PIs showed reduced CT and/or RT (Figure 4).

**Figure 5.**
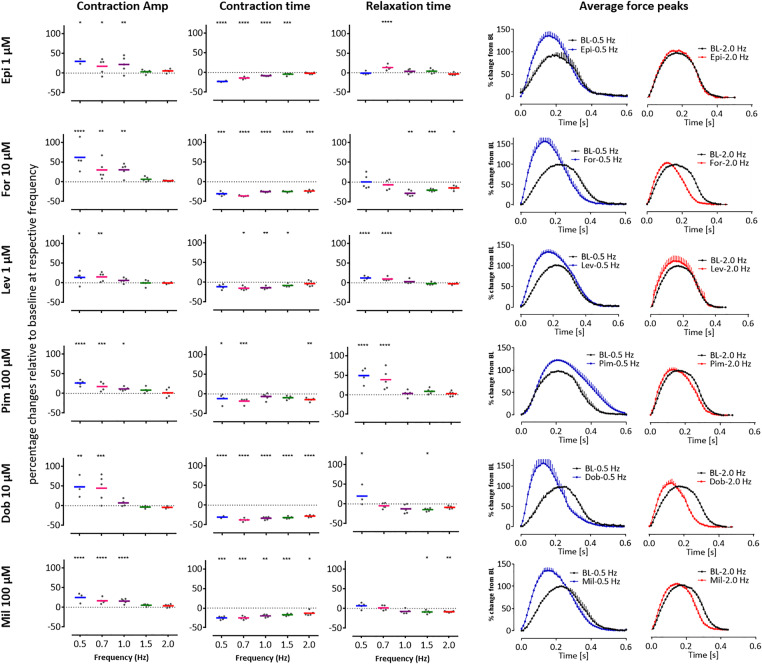
Slowed beat rate increases the predictivity of positive inotropes (PIs) in 3D engineered heart tissues (EHTs). Contraction analysis was carried out using EHTs with 6 of the PIs from the drug test set: epinephrine (Epi), forskolin (For), levosimendan (Lev), pimobendan (Pim), dobutamine (Dob), and milrinone (Mil) which had been predicted with variable accuracy (Figure 4A, web tool). In all cases, the drugs (applied as a high concentration bolus) increased contraction amplitude (CA) but only at 0.5 and 0.7 Hz, and sometimes at 1 Hz, with the largest effects seen in contraction time (CT) also often occurring at these frequencies. Scatter plots show percentage changes relative to baseline at respective frequency. Averaged peaks for force are shown for baseline (BL, black peaks) versus after treatment in EHTs paced at 0.5 Hz (blue peaks) or 2.0 Hz (red peaks). Dunnett’s stats versus baseline at respective frequency: *p < .05; **p < .01; ***p < .001; ****p < .0001.

We extended these studies to the 3D EHT platform, this time slowing spontaneous beating to < 0.5 Hz with 300 nM ivabradine, a pharmacological blocker of the “funny” *I*_f_ current (Bois *et al.*, 1996). Notably, these “slowed” EHTs could faithfully follow electrical pacing in increments from 0.5 to 2.5 Hz (Supplementary Figure 6). Whereas force-frequency relationships was negative above 1.5 Hz, it was positive between 0.5 and 1.5 Hz, evidenced by a 147% increase in force generation concurrent with significant shortening of CT and RT (Supplementary Figure 6). We therefore re-evaluated 6 of the PIs in the presence of 300 nM ivabradine with electrical pacing at 0.5, 0.7, 1.0, 1.5, and 2.0 Hz. These also included epinephrine, levosimendan, and pimobendan, which had been incorrectly predicted by EHT:R-PAT and/or EHT:ERC platform-cell combinations. Notably, for all 6 drugs, positive inotropy was evident via increases in CA but only at 0.5 and 0.7 Hz, and sometimes at 1 Hz, but never at 1.5 or 2 Hz (Figure 5). Consistent with this, shortening of CT was observed for all drugs acting via increased cAMP, with the largest changes often occurring at the lower pacing frequencies (Figure 5). Thus, in both 2D and 3D configurations, lower beating frequencies led to increased predictivity for PIs.

Finally, we considered NIs that had been incorrectly predicted (or not tested), including doxorubicin and sunitinib. Although 30 min exposure of hiPSC-CMs to these anticancer drugs altered electrophysiology, effects on contractility were predicted with variable accuracy (Table 1, web tool). We asked whether longer term exposure would affect contractility (Supplementary Figure 7). The CO:R-PAT platform-cell combination showed that exposure to the highest concentration of either drug for 24 h led to loss of contraction and cell death. Similarly, using the EHT:ERC platform-cell combination, exposure to doxorubicin ceased EHT contraction after 17.5 h, whereas exposure to 1 µM sunitinib led to a decline in CA over a 7-day period (Supplementary Figure 7). Thus, although short-term exposure to these NIs perturbed the electrophysiology of hiPSC-CMs, longer term exposure caused overt cytotoxicity and/or negative inotropy.

## DISCUSSION

Through blinded testing across multiple geographical sites, we evaluated the ability of 7 different platform-cell combinations to predict whether drugs of interest to the pharmaceutical industry were positive, negative, or neutral inotropes. We achieved this by examining contractility parameters (CA, CT, and RT), as well as using Ca^2+^ transients and/or electrophysiology to assist in decision making in some instances. Within the context of these *in vitro* models, we found that CA was the most informative parameter for NIs. Particularly after refinement involving slowing of beat rate to below 1 Hz (Figure 5, Supplementary Figure 6), CA was highly informative of PIs in the EHT system but far less so in the 2D systems. Although contraction- and relaxation-time provided confirmatory readouts for PIs in 3D EHTs, these parameters typically served as the primary source of predictivity in 2D (Figure 4), especially where the mode of action involved cAMP signaling.

We propose that an efficient way to predict the inotropic effect of drugs would be first to conduct acute (30 min) testing in hiPSC-CMs. Spontaneous beat rates should be < 1 Hz, which can be achieved by modifying the culture medium, using pharmacological blockage with ivabradine, and/or selecting hiPSC-CMs with slow intrinsic rates. In EHTs, this approach unveiled a clear positive force-frequency relationship. If no changes in inotropy are detected in the acute assay, then exposure times can be increased to ≥ 24 h to determine whether the drugs have a chronic effect. These timelines were suggested by GSK (ie, ≤ 30 min considered as acute; ≥ 24 h as chronic), which proved to be a useful approach because it allowed a predictive accuracy of 85% in 2D monolayers and 93% in 3D EHTs.

In reaching these refined conditions, we noted greater importance of the cell preparation protocol, testing conditions, methods of measuring contractility, and 2D versus 3D than the cell type used, which was not necessarily expected. There were differences in the purity/composition of the different cell types and in their baseline electrophysiological characteristics, which partly reflects their maturity state at single cell level (Supplementary Table 3). However, there was not an obvious correlation between these differences and predictivity. For example, initial testing of the same cell type (Pluricyte hiPSC-CMs) on 2 platforms (TTM and CO) gave different accuracies (78% vs 56%). The same was true for R-PAT hiPSC-CMs on the EHT and CO platforms (67% vs 44%).

Modifying both the culture environment (by including a protein source that caused a beat rate to be slowed, and signal-to-noise ratio and data variability to be reduced) and the drug exposure conditions (by including both acute chronic testing for ≤ min and ≥ 24 h, respectively) for the CO:R-PAT combination allowed the accuracy of prediction using this cell line to be increased to 85%. It is possible that further improvements could be made, whilst simultaneously shedding light on mechanism of action. For example, analysis of data during different stages of relaxation identified positive lusitropy for dobutamine (at 50% relaxation) and late relaxation deficit for ivabradine (at 80% relaxation). During the blinded phase, we also correctly predicted that drug rank 14 was omecamtiv mecarbil on account of the unusual response of increased CT (approximately +40%) without convincing evidence for increased CA (Malik *et al.*, 2011). This shows the value of applying pharmacological knowledge to drug responses in hiPSC-CMs to derive mechanistic information out of multiparametric assessments, in this case combined evaluation of CA, CT, and RT in hiPSC-CMs configured as 3D EHTs, in this case combined evaluation of CA, CT, and RT in hiPSC-CMs configured as 3D EHTs.

Another consideration is the difference between single cells, continuous monolayers, and 3D engineered constructs. The single rabbit cardiomyocyte assay used near-physiological rates of stimulation (2 Hz) and subphysiological rates (1 Hz or lower) may have improved scope to detect PIs. In adult heart preparations, a common feature of PI interventions that raise intracellular Ca^2+^ is the occurrence of spontaneous diastolic SR Ca^2+^ release. This phenomenon is linked to negative inotropy and arrhythmic behavior in single cells and intact myocardium (Allen *et al.*, 1985; Hess and Wier, 1984). In this study, spontaneous diastolic Ca^2+^ release and associated diastolic shortening was observed in isolated rabbit myocytes in response to β-adrenoreceptor stimulation and drugs that raise cAMP directly (eg, forskolin), but this phenomenon was not reported in any of the hiPSC-CMs platforms. This is consistent with the minimal involvement of the SR in excitation-contraction coupling typically seen in embryonic cardiomyocytes and hiPSC-CMs (Knollmann, 2013); in this context, the PI effect from cAMP arises mainly from cAMP-mediated stimulation of L-type Ca^2+^ current.

Out of necessity, preparations of native adult CMs are dispersed and seeded as cultures of single cells. It is known that variance in cell density influences electrophysiological parameters (Du *et al.*, 2015), particularly within, and between, preparations of single cell preparations, including hiPSC-CMs (Mosqueira *et al.*, 2018). Therefore, although the structural and function (eg, prevalent SR) maturity of adult native adult CMs from rabbit is an advantage over hiPSC-CMs, this is offset by the high level of heterogeneity of single cell preparations, which reduces the discrimination power. Although some of the differences will be due to the physiology of rabbit CMs relative to human CMs, we have also seen higher variability in human atrial trabeculae as compared with hiPSC-EHT; hence, there are separate challenges in using native cells. It is for these reasons we elected to use hiPSC-CMs within 2D or 3D syncytium, where individual cell-to-cell variability is averaged due to mechanical and electrical coupling. In addition, several studies have shown previously that the EHT format compared with standard 2D culture favors maturation in terms of MDP and upstroke velocity (Lemoine *et al.*, 2017, 2018), structure (Mannhardt *et al.*, 2016), and metabolic preference (Ulmer *et al.*, 2018).

Blinded analysis was done using serum-/protein-free medium, with the intention of avoiding protein-drug binding that might blunt the responses within the *in vitro* system. However, at least in the CO:R-PAT platform-cell combination, serum-/protein-free medium was a hindrance, leading to high spontaneous beat rates, and poor signal-to-noise ratio ratios. These issues were abrogated by using protein-containing medium (RPMI-B27), which enhanced the accuracy of predicting PIs on the CO:R-PAT platform from 0% to 75%. This indicates that, for this purpose, the benefits brought by the protein-containing medium outweigh the concerns of drug binding and batch-to-batch variations of protein ingredients. Nevertheless, voltage-sensitive dyes, such as FluoVolt, interact with proteins and reduce the signal-to-noise ratio, which make simultaneous recording of contraction and voltage challenging, although this may be overcome by measuring extracellular voltage.

Treatment of hiPSC-CMs with doxorubicin for 30 min caused changes in electrophysiology (eg, triangulation, see web tool) and hence gave a distinctive response compared with NEs such as acetylsalicylic acid. Nevertheless, this acute exposure did not always cause changes in inotropy, which shows the importance of examining an appropriate concentration range and/or exposure times of drugs. Increasing exposure time to approximately 1–7 days unveiled doxorubicin and sunitinib as NIs, consistent with the extended timescale over which cardiac toxicity presents clinically and is in line with data from a previous study using TKI-inhibitors (Jacob *et al.*, 2016). Interestingly, with 24 h of doxorubicin treatment, the CO:R-PAT platform-cell combination appeared to show a trend of positive inotropy from 1 to 30 µM (increased CA; decreased CT) but cell death at 10 µM. This aligns with reports indicating the effects of doxorubicin are complex in that transient positive inotropy is followed by robust negative inotropy at higher concentrations (Kim *et al.*, 1980; Vanboxtel *et al.*, 1978; Wang and Korth, 1995).

The levels of accuracy of up to 93% in predicting drug-induced changes in contractility in human hearts under these refined conditions are favorable relative to those from animal models (Lawrence *et al.*, 2008; Valentin *et al.*, 2009). They are also comparable with data using 3D cardiac microtissues containing hiPSC-CMs, cardiac endothelial cells, and cardiac fibroblasts, which correctly predicted 23 of 29 (85%) inotropes across a nonblinded panel of compounds (Pointon *et al.*, 2017). These findings are encouraging, but it is not always straightforward to translate hiPSC-CM-related parameters of CA, CT, RT, and chronotropy (beat rate) to clinically relevant data. We have suggested that data derived from hiPSC-CMs on CA is informative for NIs in all cell-platforms combinations and for PIs in the 3D EHT platform. However, in the 2D systems, CT, and RT may be informative for PIs. We have considered different explanations for this observation. If 2D systems are less adept at showing an increase in CA, then decreases in CT (clinotropy; also expressed as an increase in d*F*/d*t*) or decreases in RT (lusitropy; also expressed as an increase in −d*F*/d*t*) become more important. By considering p*F*/d*t* values, which can change independently of the peak force, there is alignment with the effects seen *in vivo*, which are often expressed as a change in d*P*/d*t*_max_ or −d*P*/d*t*_min_. An alternative explanation is that, although this is not automatically intuitive, it may be a quirk of hiPSC-CMs; as a model system, there are limitations and adaptations in thinking need to be made. Nevertheless, the robustness of this notion will need to be tested on a wider range of drugs to determine whether these *in vitro* parameters are relevant to *in vivo* cardiac physiology.

Effects on cardiac contractility *per se* may not indicate or predict a clinically relevant detrimental effect on the heart. For example, cardio-active L-type calcium channel blockers are used for treatment of hypertension. Additional “structural” endpoints, such as mitochondrial membrane permeability (Δ*ψm*), endoplasmic reticulum integrity, contractile filament expression/organization, ATP depletion, and cardiac troponin levels in hiPSC-CM-based assay platforms may add interpretive value to functional changes (Sala *et al.*, 2018). A limitation of our study was that some modes of action were not represented in the drug panel, such as the SERCA activator CDN1163 (Dahl, 2017; Kang *et al.*, 2016), or the myosin inhibitor blebbistatin (Kovacs *et al.*, 2004), and these would be interesting to include in blinded assays in the future.

In our study, we looked for trends or significant changes in the parameters measured, but did not apply a cut off on how much change was considered meaningful (eg, > 15% change *and* statistical significance). Whether analysis can be modified in the future is for consideration but would need to be done with care. The NE drugs, enalaprilat, tolbutamide, and pravastatin caused changes of 19%–33% in CA on the EHT platform and were incorrectly predicted as PIs. However, these magnitudes of changes were similar or greater than those recorded for PIs, and so adding thresholds on CA would not have improved prediction and could have been detrimental. Relationship to FTPC was not a strong association either, with the maximum changes of the incorrectly predicted NEs varying from 3-fold below the FTPC (tolbutamide) to > 100-fold above (enalaprilat, pravastatin). This also underscores the importance of technical precision and inclusion of time controls in each single experiment, particularly in strategies employing cumulative concentration-response analyses which take time.

These observations likely reflect the complexity of drug-cell interactions, which means differential effects can occur dependent on the concentration and categorizing as PI, NI, and NE was difficult in some cases. Inotropic effects of high concentrations of sulphonylureas, such as tolbutamide, have been reported for *in vitro* systems (Huupponen, 1987). Angiotensin converting enzyme (ACE) inhibitors, such as enalaprilat, lead to bradykinin accumulation and thereby lead to bradykinin B1 receptor activation, which elevates intracellular Ca^2+^ and causes a positive inotropic effect (Ignjatovic *et al.*, 2002). Similarly, phentolamine is a neutral inotrope at free plasma concentrations of approximately 2.5 µM in patients (Wallis *et al.*, 2015), but at concentrations above 10 µM phentolamine causes negative inotropy via modulation of fast sodium and L-type calcium channels (Rosen *et al.*, 1971; Sada, 1978). This is consistent with our data, indicating that there are multiple pharmacological effects of this compound. For ivabradine the concentrations provided were much higher than the FTPC, which can lead to off-target effects of poorly selective drugs (Choi *et al.*, 2016). In some cases, such as zimelidine, the difficulty arose due to lack of robust *in vitro* data within the literature (Forsberg and Lindbom, 2009; Lindbom and Forsberg, 1981; Naranjo *et al.*, 1984). Care must therefore be used when (1) selecting test compounds by primary pharmacology and (2) using *in vitro* assay paradigms to predict *in vivo*, clinically relevant cardiotoxicity, either when used as a “screening assay” to rank compounds or as a reflex investigative assay eg, following detection of cardiotoxicity in animal toxicology studies.

Because PIs are cardio-active drugs, it might be expected that the FTPC aligns with responses from hiPSC-CMs. This was true for the EHT platform, where significant responses were within, or close to, the FTPC. However, both the pharmacologic or statistical sensitivity of 2D hiPSC-CM cultures and isolated single rabbit cardiomyocytes were considerably lower. Two explanations may account for these observations. First, the greater stability of CMs in 3D constructs permitted cumulative dosing of EHTs within the same well, which created tighter datasets than the 2D systems, where parallel dosing in separate well was required. Second, in contrast to 2D monolayers that evaluated hiPSC-CM movement in unloaded conditions, the EHT platform measures force of contraction under loaded conditions (Eder *et al.*, 2016). This facilitates maturation and Frank-Starling mechanisms for force generation are followed, leading to higher basal tone and cAMP signaling (Huupponen, 1987; Uzun *et al.*, 2016), which are important modes of action of the PIs epinephrine, forskolin, and milrinone. Consistent with this, positive inotropy of milrinone was shown in “Biowire II” tissue engineered constructs, which also places hiPSC-CMs under loaded conditions to facilitate maturation (Zhao *et al.*, 2019).

To measure whole heart function and its integration with neurohormonal or hemodynamic feedback, numerous assessment parameters are used, including *P*_max_, d*P*/d*t*_max_ (max rate of change if pressure) and left ventricular ejection fraction (Guth *et al.*, 2015). Atenolol, a selective β1-adrenoceptor antagonist, requires intact sympathetic innervation and is an NI in the clinic but shows NE in hiPSC-CMs (Kaumann and Blinks, 1980; Lemoine *et al.*, 1988). Similarly, the mode of action for clonidine is through presynaptic alpha-adrenoceptors on sympathetic neurons with the consequence of reduced sympathetic drive and its effect on heart function (reduced heart rate and force) (Jarrott *et al.*, 1979; Kleiber *et al.*, 2017), but these effects would not be detected in a “cardiomyocyte only” model. Predictive screening will also benefit from inclusion of auxiliary cell types, such as neural lineages to enable evaluation of drugs that work via the neurohormonal system.

Other issues that need to be considered for the future include throughput, user variability, cell availability, cost, and batch-to-batch variability. Although the TTM platform allowed simultaneous measurement of contraction, Ca^2+^ handling, and voltage, the low throughput meant that only 10 drugs were evaluated in 1 hiPSC-CM line. User variability has been noted in other studies. For example, variations in TdP predictability across different sites using identical instruments were reported in the CiPA study^13^, and so could be a factor in the site to site differences we observed. Cost was another factor, wherein each drug assessed typically required 1.6 million (40 wells in a 96-well plate) and 5 million (5 EHTs) hiPSC-CMs, corresponding to up to approximately $3000 per drug at current prices. For these reasons, most of the assays in this report were done using hiPSC-CMs produced “in house,” where cost for 1.6 or 5 million cells is approximately $16 or $50, respectively, albeit without the same level of quality control of commercial cells. Nevertheless, for both in house and commercial cells, batch-to-batch variation meant that the drug assays need to be repeated. These issues become more prominent when larger tissue engineered constructs are used. For example, “heart-in-a-jar” technologies, produce miniature 3D engineered electro-mechanically coupled cardiac organoid chamber that mimic pumping action similar to natural heart but require 10 million hiPSC-CMs (Li *et al.*, 2018). Thus, it is encouraging that scaled production of hiPSC-CMs is now becoming relatively routine and so costs should decrease in the near future.

Altogether, this study suggests that, even in their current status of technology evolution, hiPSC-CMs cultured in 2D and 3D may have value to predictive safety pharmacology. Given the high resource and costs involved with drug development, even modest improvements in the pipeline could have large socioeconomic and 3R benefits. More than 6000 putative medicines are in preclinical development, using millions of animals at an annual total cost of $11.3Bn, and so each percentile reduction equates to approximately $100 M. Similarly, reducing drug attrition in phase 1 clinical trials by 5% could reduce development costs by 5.5%–7.1%. Building on our work with further improvement and validation studies, conducted in a blinded manner, will facilitate uptake of hiPSC-CMs as a routine tool in safety pharmacology.

## MATERIALS AND CORRESPONDENCE

Indicate the author(s) to whom correspondence and material requests should be addressed. General enquiries should be to C.D., whereas specific enquiries should be directed as follows: CO:Rb (G.L.S), TTM:hiPSC-CM (C.L.M.), CO:hiPSC-CM (C.D.), and EHT:hiPSC-CM (A.H.).

## SUPPLEMENTARY DATA

Supplementary data are available at *Toxicological Sciences* online.

## Supplementary Material

kfaa058_Supplementary_Data
