# Supplementary material for: Blinded, Multicenter Evaluation of Drug-induced Changes in Contractility Using Human-induced Pluripotent Stem Cell-derived Cardiomyocytes
Source: Toxicol Sci. 2020 May 18;176(1):103–23. doi: 10.1093/toxsci/kfaa058 (PMC7357169; doi:10.1093/toxsci/kfaa058)
Supplement: kfaa058_Supplementary_Data [file kfaa058_supplementary_data.docx]

##### **Title:**

**Blinded, multi-centre evaluation of drug-induced changes in contractility using human induced pluripotent stem cell-derived cardiomyocytes**

##### **Single sentence summary:**

hiPSC-cardiomyocytes predict with 93% accuracy effect of drugs on heart contractility, validating use in preclinical drug development and safety assessment

## **Authors:**

Umber Saleem^1†^, Berend J van Meer^2†^, Puspita A Katili^3^, Nurul A N Mohd Yusof^3^, Ingra Mannhardt^1^, Ana Krotenberg Garcia^2^, Leon Tertoolen^2^, Tessa de Korte^2,4^, Maria LH Vlaming^4^, Karen McGlynn^5^, Jessica Nebel^1^, Anthony Bahinski^6^, Kate Harris^7^, Eric Rossman^6^, Xiaoping Xu^6^, Francis L Burton^5,8^, Godfrey L Smith^5,8^, Peter Clements^9^, Christine L Mummery^2,10^, Thomas Eschenhagen^1^, Arne Hansen^1^, Chris Denning^3*^

## **Affiliations:**

^1^ Department of Experimental Pharmacology and Toxicology, University Medical Center Hamburg Eppendorf, Martinistraße 52, 20246 Hamburg, and DZHK (German Center for Cardiovascular Research), partner site Hamburg/Kiel/Lübeck, Germany. Email: [u.saleem@uke.de](mailto:u.saleem@uke.de); [i.mannhardt@uke.de](mailto:i.mannhardt@uke.de); [t.eschenhagen@uke.de](mailto:t.eschenhagen@uke.de); [ar.hansen@uke.de](mailto:ar.hansen@uke.de); [Jessica-Nebel@web.de](mailto:Jessica-Nebel@web.de)

^2^ Dept. of Anatomy and Embryology, Leiden University Medical Center, Einthovenweg 20, 2333 ZD Leiden, The Netherlands. Email: [B.J.van_Meer@lumc.nl](mailto:B.J.van_Meer@lumc.nl); [L.G.J.tertoolen@lumc.nl](mailto:L.G.J.tertoolen@lumc.nl); [C.L.Mummery@lumc.nl](mailto:C.L.Mummery@lumc.nl)

^3^ Dept. of Stem Cell Biology, University of Nottingham, University Park, NG7 2RD, Nottingham, United Kingdom. Email: PAK, [mzxpk@exmail.nottingham.ac.uk](mailto:mzxpk@exmail.nottingham.ac.uk); NANMY, [Nurul.MohdYusof@nottingham.ac.uk](mailto:Nurul.MohdYusof@nottingham.ac.uk); [chris.denning@nottingham.ac.uk](mailto:chris.denning@nottingham.ac.uk)

^4^ Ncardia, Galileiweg 8, 2333 BD, Leiden, The Netherlands. Email: [tessa.dekorte@ncardia.com](mailto:tessa.dekorte@ncardia.com); Marijn.vlaming@crl.com

^5^ Clyde Biosciences Ltd, Biocity Scotland, Bo’Ness Road, Newhouse, Lanarkshire, ML1 5HU, Scotland, United Kingdom. Email : [kpm@clydebio.com](mailto:kpm@clydebio.com)

^6^ GlaxoSmithKline, 1250 S. Collegeville Rd., Collegeville, PA 19426 – USA. Email: [anthony.x.bahinski@gsk.com](mailto:anthony.x.bahinski@gsk.com); [eric.i.rossman@gsk.com](mailto:eric.i.rossman@gsk.com); xiaoping.2.xu@gsk.com

^7^ NC3Rs. Gibbs Building 215 Euston Road London NW1 2BE. United Kingdom. Email: Kate. Harris@nc3rs.org.uk

^8^ Institute of Cardiovascular and Medical Sciences, University of Glasgow, G12 8QQ, Glasgow, United Kingdom. Email: [Francis.Burton@glasgow.ac.uk](mailto:Francis.Burton@glasgow.ac.uk); [Godfrey.Smith@glasgow.ac.uk](mailto:Godfrey.Smith@glasgow.ac.uk)

^9^ GlaxoSmithKline, David Jack Centre for R&D, Park Road, Ware, Hertfordshire, SG12 0DP, United Kingdom. Email: [peter.j.clements@gsk.com](mailto:peter.j.clements@gsk.com)

^10^ Dept. Applied Stem Cell Technologies, University of Twente, 7500 EA Enschede, the Netherlands. Email: [C.L.Mummery@lumc.nl](mailto:C.L.Mummery@lumc.nl)

^†^ Authors contributed equally to work; * Corresponding author: [chris.denning@nottingham.ac.uk](mailto:chris.denning@nottingham.ac.uk)

**Short title**: Drug impact on hiPSC-CMs contractility

**Supplementary Table 1. Compound selection within the CRACK-IT study**. Eight training, non-blinded “Tier 1 (T1) training set” compounds with known positive or negative inotropic effects were selected to standardise the working practices across the platform-cell combinations within the study. Twenty eight “Tier 2 (T2) test set” compounds were chosen for the blinded phase of the study, of which 18 were positive inotropes (PI) or negative inotropes (NI) and span the main modes of action known to alter contractility responses of cardiomyocytes. The remainder were considered as no effect drugs (NE, no effect on cardiac contractility). A 5 x ½ log range was chosen based on the literature and/or free therapeutic plasma concentration (FTPC). Abbreviations: n.d, not defined; n.a, not applicable; PC, positive chronotropy; PI, positive inotropy; PCl, positive clinotropy; PL, positive lusitropy; NC, negative chronotropy; NI, negative inotropy; NCl, negative clinotropy; NL, negative lusitropy; inotropy is defined as change in peak force, clinotropy as change in time-to-peak force (generally from 20% above baseline to peak and “positive” being an abbreviation) and lusitropy as change in relaxation time (time from peak force generally to 80% relaxation and “positive” being an abbreviation); EC_50_, half maximal effective concentration; IC_50_, half maximal effective inhibitory concentration; GP, guinea pig; hERG, human eag-related gene; NCX, Na^+^/Ca^2+^ exchanger; RR, blood pressure; RyR2, ryanodine receptor 2; SR, sarcoplasmic reticulum; * indicates lack of clarity in literature on inotropic effect; # in spontaneously contracting preparations.

| **Drug** | **Use** | **PI, NI, NE** | **Main mechanism of action** | **Expected cardiac**  **effects in vivo** | **Expected effect on contractility in isolated cardiomyocytes/heart muscles** | **Cellular mechanism**  **of inotropic effect** |  | **Concentration range applied (µM)** | **FTPC (μM)** | **Refs** |
| --- | --- | --- | --- | --- | --- | --- | --- | --- | --- | --- |
| Isoprenaline | T1 | PI | β1- and β2-adrenoceptor agonist | PI, PC | PI, PC#, PCl, PL  EC_50_ 0.01 µM | Increase in cAMP, Ca^2+^ influx,  SR Ca^2+^ uptake and SR load |  | 0.0003-0.1 | 0.0095 | (Reyes et al. 1993) |
| Digoxin | T1 | PI | Na^+^/K^+^ ATPase inhibitor | PI, arrhythmias | PI+ PL (< 1 µM, slowly developing, t_1/2_ conc.-dependent)  NI + diast. shortening (>1 µM) | Na^+^/K^+^ inhibition,  Ca^2+^ increase via NCX |  | 0.01-10 | 0.00075-0.0015 | (Lullmann and Ravens 1973) |
| Bay K 8644 | T1 | PI | L-type Ca^2+^ channel agonist | PI (indirect NC because of RR increase) | PI, NL | Increase in Ca^2+^ influx |  | 0.03-3 | n.d. | (Aass et al. 1988) |
| EMD 57033 | T1 | PI | Calcium sensitizer | PI | PI, NL, EC_50_ ~5 µM, diastolic shortening at high conc. | Myofilament sensitization  to Ca^2+^ |  | 0.01-30 | 16 µM | (Hajjar et al. 1997), (de Zeeuw et al. 2000) |
| Caffeine | T1 | PI | Opener of RyR2 (at high concentration) | PI or NI o NE | Small PI and PL at 100 µM, transient large PI, NL at >10 mM | Opening of RyR2 |  | 100-10,000 | 20-40 | (Chaban et al. 2017) |
| Nifedipine | T1 | NI | L-type Ca^2+^ channel blocker (dihydropyridine) | Small NI (direct) overridden by adrenergic activation, PC (indirect) | NI, IC_50_ 0.1 µM | Decreased Ca^2+^ influx |  | 0.003-0.3 | 0.02 | (Angus et al. 2000) |
| Ryanodine | T1 | NI | Inhibitor of RyR2 | n.d. | NI, NCl, species dependent biphasic, IC_50_ ~0.01 µM | Decreased SR Ca^2+^ release |  | 1-30 | n.d. | (Sutko and Willerson 1980), (Fedorov et al. 2002) |
| Thapsigargin | T1 | NI | Inhibitor of SR Ca^2+^ ATPase | n.d. | Heart muscle: NI ~50% at 500 µM, NL ~20% at 500 µM  Isolated myocytes: ~90% NI at 0.1 µM | Decreased SR Ca^2+^ uptake  and load |  | 0.3-30 | n.d. | (Kirby et al. 1992), (Baudet et al. 1993) |
| Epinephrine | T2 | PI | Non-selective α1-, β1- and β2-adrenoceptor agonist | PI, PC | PI, PC#, PCl, PL  EC_50_ 0.1 µM | Increase in cAMP, Ca^2+^ influx, SR Ca^2+^ uptake and SR load |  | 0.01 - 1 | 0.0002-0.050 | (Molenaar et al. 2007), (Wortsman et al. 1984) |
| Forskolin | T2 | PI | Adenylyl cyclase stimulator | PI, PC | PI, PC#, PCl, PL  EC_50_ 5-10 µM | Increase in cAMP, Ca^2+^ influx,  SR Ca^2+^ uptake and SR load |  | 0.1 - 10 | 0.012-0.024 (colforsin) | (Bristow et al. 1984), (Kikura et al. 2004) |
| Levosimendan | T2 | PI | PDE3 inhibitor (IC_50_ 25 nM)  Calcium sensitizer  K_ATP_ channel agonist | PI, PC (more heart rate increase and RR decrease than dobutamine) | Small PI (~50%), PC#, PCl+PL (14%),  EC_50_ ~0.1 µM | Increase in cAMP and myofilament sensitization to Ca^2+^ |  | 0.01 - 1 | 0.026-0.35 | (Boknik et al. 1997), (Hasenfuss et al. 1998), (Mebazaa et al. 2007), (Papp et al. 2012), (Orstavik et al. 2014), (Abi-Gerges et al. 2013), (Puttonen et al. 2008) |
| Pimobendan | T2 | PI | Calcium sensitizer  PDE inhibitor (partial) | PI, small PC | PI, small PC#, PL and NL at low/high conc.,  EC_50_ 34 µM | Increase in cAMP and myofilament sensitization to Ca^2+^ |  | 1 - 100 | 0.005-0.01 | (Honerjager et al. 1984), (Berger et al. 1985), (Chu et al. 1995) |
| Dobutamine | T2 | PI | α1-, β1- and β2  adrenoceptor agonist | PI, PC, less HR-stimulation than epinephrine | PI, PC#, PCl, PL.  EC_50_ 1-3 µM | Increase in cAMP, Ca^2+^ influx, SR Ca^2+^ uptake and SR load |  | 0.1 - 10 | 0.07-1 | (Ishihata et al. 1988), (Brown et al. 1987), (Mahoney et al. 2016) |
| Milrinone | T2 | PI | PDE3 inhibitor | PI, PC (more heart rate increase and RR decrease than dobutamine) | Small PI, PC#, PCl, PL (smaller than ISO)  EC_50_ 60 µM | Increase in cAMP, Ca^2+^ influx, SR Ca^2+^ uptake and SR load |  | 1-100 | 0.1-0.15 | (Brown et al. 1986), (Bailey et al. 1994) |
| Omecamtiv mecarbil | T2 | PI | Cardiac specific  myosin activator | PI, prolonged syst. ejection time, small decrease in heart rate | PI, NCl, NL, diastolic shortening (dog myocytes).  EC_50_ 1 µM | Increased number of  myosin heads binding  to actin, no affection  of Ca^2+^ transient |  | 0.01 - 1 | 0.05 -0.42 | (Cleland et al. 2011), (Planelles-Herrero et al. 2017), (Horvath et al. 2017), (Teerlink et al. 2011), (Liu et al. 2016) |
| Terbutaline | T2 | PI | β2-adrenoceptor  agonist | Small PI and PC | PI, PC#, PCl, PL.  EC_50_ 3 µM | Increase in cAMP, Ca^2+^ influx, SR Ca^2+^ uptake and SR load |  | 0.1 - 10 | 1.3 | (Schafers et al. 1994), (Dyreborg et al. 2016) |
| Verapamil | T2 | NI | L-type Ca^2+^ channel blocker,  hERG blocker | NI (direct) partially masked by adrenergic activation (indirect), NC or none, ND (direct) | NI, IC_50_ 0.1 µM | Decreased Ca^2+^ influx |  | 0.01 - 1 | 0.05 | (Angus et al. 2000), (Giacomini et al. 1984) |
| Doxorubicin | T2 | NI | Impairs Ca^2+^ transport mechanisms in sarcoplasmic reticulum | NI, time course of cardiotox dose-dependent | PI+NCl and reduced post-rest potentiation at 30 µM (GP), but strong NI at 1000 µM (GP, rat); rate-dependent | Complex, including increased  mitochondrial ROS |  | 0.1 - 10 | 1-2 | (Temma et al. 1993), (Hofling and Bolte 1981), (Matsushita et al. 2000), (Danesi et al. 1999) |
| Sunitinib | T2 | NI | Multi-targeted TK inhibitor | NI, NC at high doses, chronic cardiotox | NI, IC_50_ 10 µM | Complex |  | 0.1 - 10 | 0.003 | (Mooney et al. 2015), (Abi-Gerges et al. 2013), (Bello et al. 2006) |
| Citalopram | T2 | NI | SSR inhibitor, hERG & L-type Ca^2+^ channel inhibitor | None (at therapeutic doses), higher risk of TdP | NI at 30 µM (-57% in hEHT, -38% in human atria) | Decrease in Ca^2+^ influx |  | 1 - 100 | 0.05 | (Witchel et al. 2002), (Mannhardt et al. 2017), (Rao 2007) |
| Itraconazole | T2 | NI | Triazole antifungal, inhibitor of Na^+^ channels and mitochondrial voltage-dependent anion channel 1 | NI | NI (-30% at 0.3 µM; rabbit heart) | Decreased Na^+^ influx (?) |  | 0.1 - 10 | 0.00086 (higher in tissues) | (Head et al. 2015), (Qu et al. 2013), (Bellmann 2007) |
| Sorafenib | T2 | NI | Multi-targeted kinase inhibitor | None or NI at high doses, delayed cardiotox | None (feline and canine CM, but cell death), NI (rat heart) | n.d. |  | 0.1 - 10 | 0.03 (higher tissue levels) | (Henderson et al. 2013), (Duran et al. 2014), (Abi-Gerges et al. 2013), (Kim et al. 2013) |
| Ivabradine* | T2 | NI | I_f_ inhibitor | NC (therapeutic) | None, NC at >10 µM (spontaneously beating preps), NIE (-13/-60% in guinea pig atria/PM, -38% human trabeculae at 100 µM); PI in mouse left atria and 30% of human trabeculae | Decrease in Ca^2+^ transient peak |  | 0.1 - 10 | 0.01-0.1 | (Perez et al. 1995), (Boldt et al. 2010), (Bois et al. 1996), (Mesirca et al. 2014), (Choi et al. 2016) |
| Flecainide | T2 | NI | Na^+^ channel blocker,  hERG blocker | NI | NI, IC_50_ 0.3 µM | Decreased Na^+^ influx (?) |  | 0.1 - 10 | 0.2-0.4 | (Abi-Gerges et al. 2013), (McQuinn et al. 1988) |
| Phentolamine | T2 | NI | Non-selective α adrenoceptor antagonist, Na^+^ and Ca^2+^ channel blocker at high conc. | PC (indirect) | NI, IC_50_ ~20 µM | Decreased Na^+^ and Ca^2+^  influx |  | 1-100 | 2.25 | (Sada 1978), (Rosen et al. 1971) |
| Zimelidine* | T2 | NI | SSR inhibitor | None (at ther. doses), QT and QRS widening at high doses | n.d. | n.d. |  | 1 - 100 | 0.78 | (Lindbom and Forsberg 1981), (Forsberg and Lindbom 1983), (Naranjo et al. 1984) |
| Acetylsalicylic acid | T2 | NE | Cyclooxygenase inhibitor | None | None | n.a. |  | 1 - 100 | 0.3-2 | (Abi-Gerges et al. 2013) |
| Atenolol | T2 | NE | β1>β2 adrenoceptor  antagonist | NI, NC, ND | None (in absence of agonist) until 3000 µM  (in contrast to e.g. propranolol) | n.a. |  | 0.1 - 10 | 1 | (Lemoine et al. 1988), (Kaumann and Blinks 1980) |
| Captopril | T2 | NE | ACE inhibitor | None | None | n.a. |  | 1-100 | 1-2 | (Abi-Gerges et al. 2013), (Giudicelli et al. 1987) |
| Glibenclamide | T2 | NE | K_ATP_ channel antagonist | None or PI in ischemia | None; PI in ischemia or presence of K_ATP_ opener; NI in adrenalectomized dogs, NE in isolated dog heart trabeculae | n.a. |  | 0.1 - 10 | 0.02-0.06 | (Satoh et al. 1990), (Murakami et al. 1992), (Pogatsa and Dubecz 1977), (Rydberg et al. 1997) |
| Enalaprilat | T2 | NE | ACE inhibitor | None | None | n.a. |  | 1 - 100 | 0.12 | (Stage et al. 2017) |
| Clonidine | T2 | NE | α2-adrenoceptor agonist | NI, NC | None | n.a. |  | 0.01 - 1 | 0.002-0.004 | (Jarrott et al. 1979), (Kleiber et al. 2017) |
| Paracetamol | T2 | NE | Prostaglandin synthesis inhibitor | None | None | n.a. |  | 10 - 1000 | 50 | (Marks et al. 2012), (Kamali et al. 1993), (Brown et al. 1992) |
| Tolbutamide | T2 | NE* | K_ATP_ channel antagonist, weak adenylyl cyclase stimulator | None or PI (dogs, men) | None (canine CM) or small PI (rabbit atria, rat heart), EC_50_ ~30 µM | Increase in cAMP (?) |  | 1 - 100 | 20-30 | (Levey et al. 1971), (Lasseter et al. 1972), (Abi-Gerges et al. 2013), (Whiting et al. 1981) |
| Pravastatin | T2 | NE | HMG CoA reductase inhibitor | None | None | n.a. |  | 1 - 100 | 0.018 | (Costantine et al. 2016) |
| Sildenafil | T2 | NE | PDE5 Inhibitor | None or PI+PC (indirect) | None | n.a. |  | 0.3 - 30 | 0.02 | (Sugiyama et al. 2001), (Walker et al. 1999) |

**
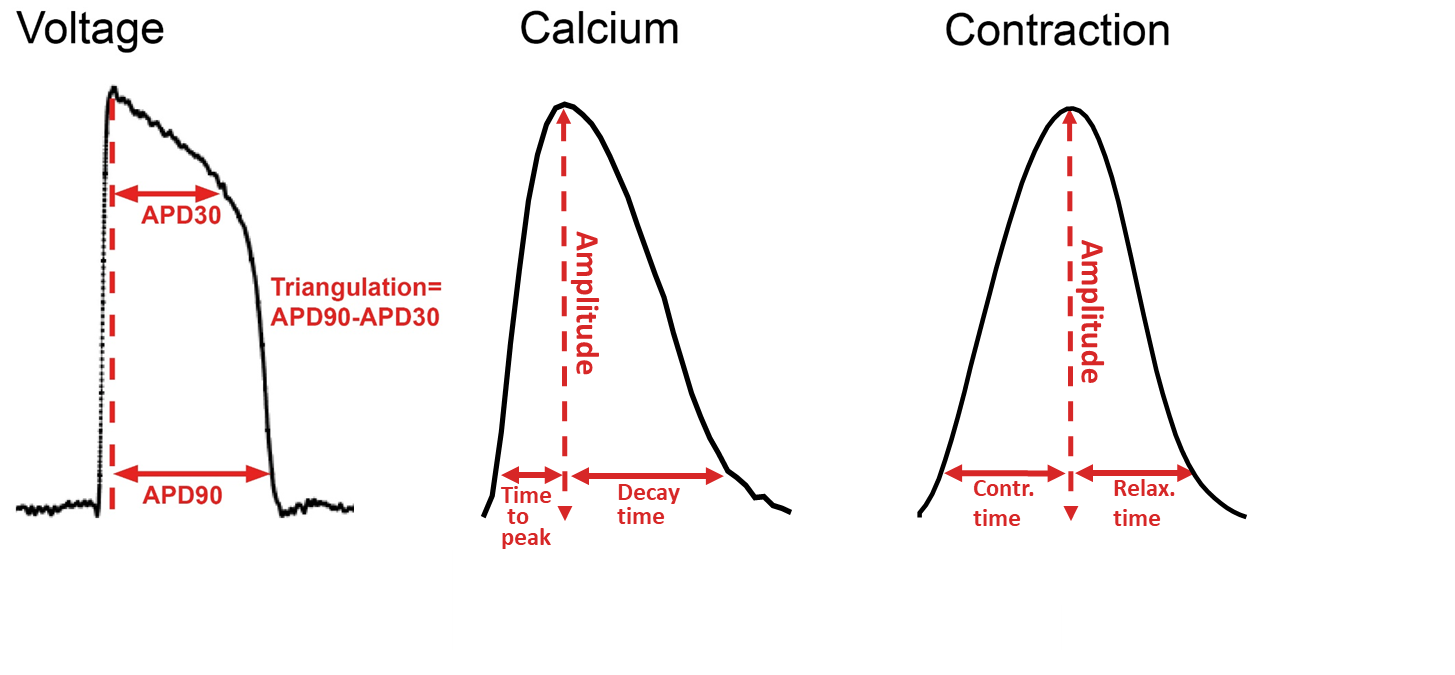
**

**Supplementary Figure 1: Schematic description of the parameters used to quantify drug effect in hiPSC-CMs.** Data were calculated using the *MUSCLEMOTION* algorithm. APD, action potential duration.

**
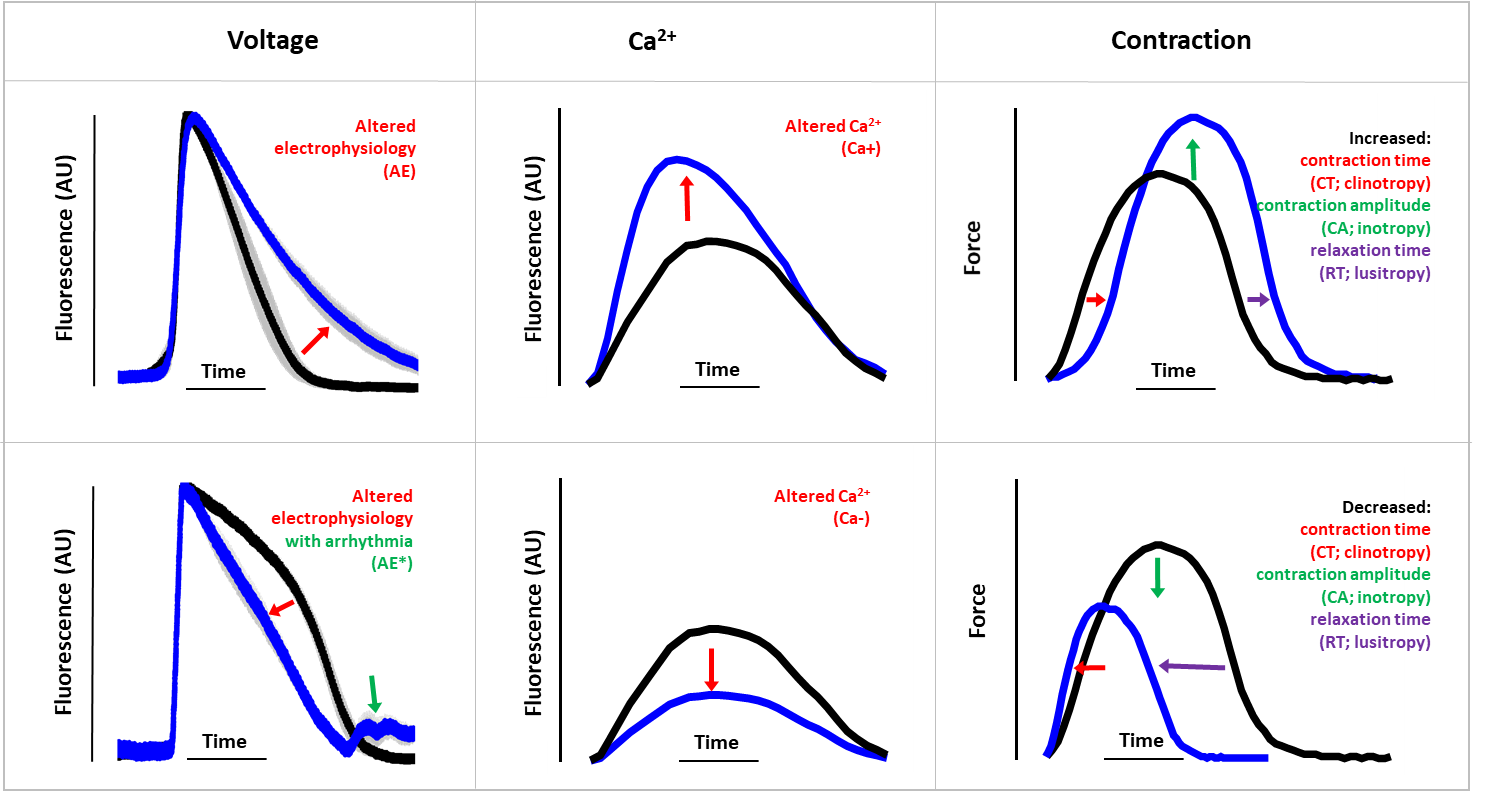
**

**Supplementary Figure 2: Terminology used in evaluation of responses**. Black traces are baseline readings, while blue are after treatment. The colour of the arrows and text matches to indicate the type of change occurring.

**
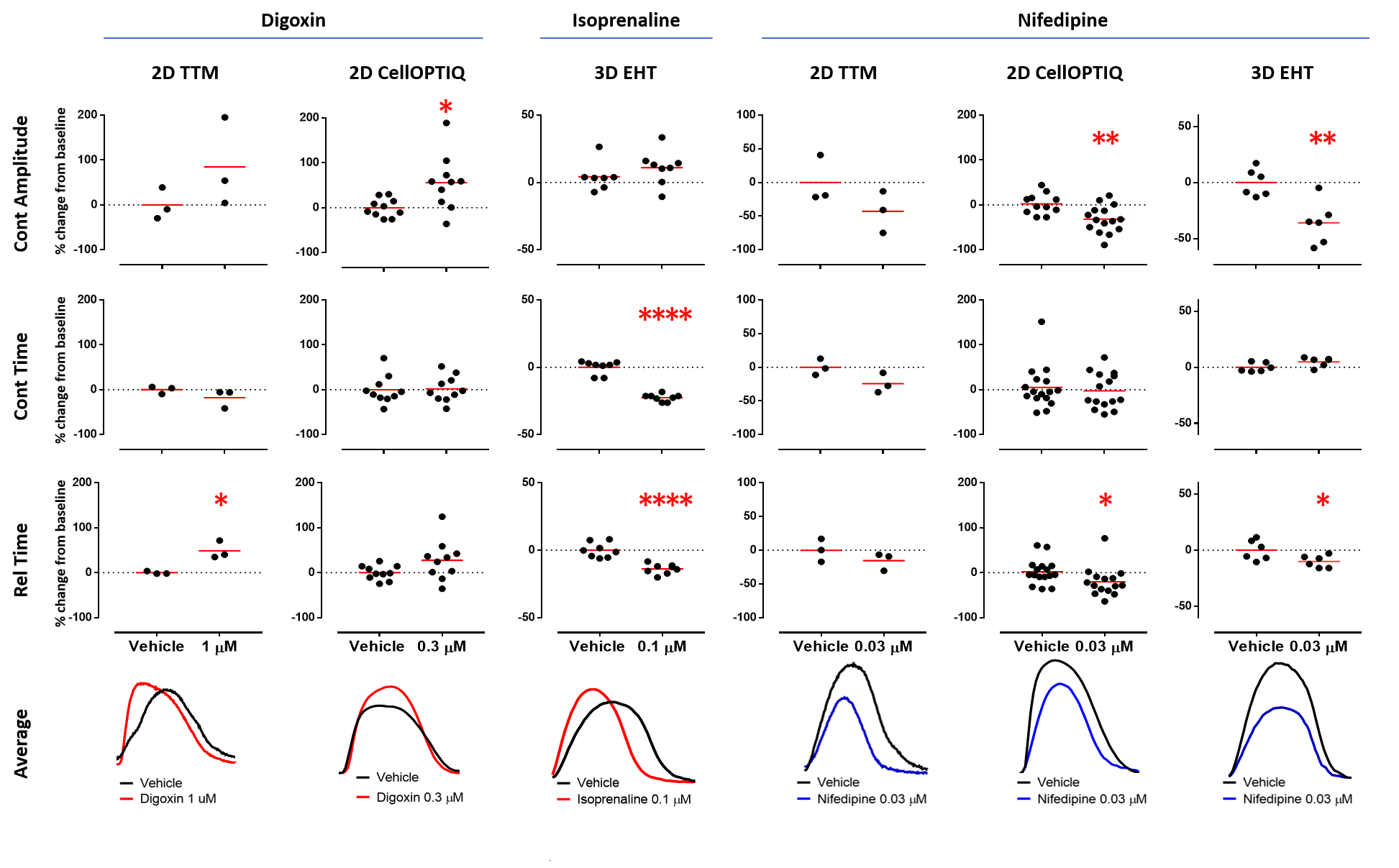
**

**Supplementary Figure 3: Training sets to unify drug testing procedures.** Representative contractility data are shown for positive (digoxin, 2D; isoprenaline, 3D) and negative (nifedipine 2D, 3D) inotropes evaluated in hiPSC-CMs using the TTM (Triple Transient Measurement), CellOPTIQ® and EHT (engineered heart tissue) platforms. The percentage change in drug treated samples is relative to their respective vehicle control. Unpaired T-test. * p<0.05; ** p<0.01; *** p<0.001; **** p<0.0001.

**Supplementary Table 2: Comparison and unification of standard operating procedures for TTM, CO and EHT platforms.** In house means the conditions or assays used for hiPSC-CMs derived from in house hiPSC lines using in house differentiation methods. Commercial means use of Pluricyte and/or iCell^2^ hPSC-CMs that were purchased from Ncardia and Cellular Dynamics International, respectively.

|  | Protocol | Triple Transient Measurement (TTM) | CellOPTIQ^®^ (CO) | Engineered Heart Tissues (EHTs) |
| --- | --- | --- | --- | --- |
| *Starting conditions* | Format | 96-well plate | | - 1 million hiPSC-CMs / EHT |
|  | Age | - Thawing + 5-7 days | - Thawing + 5-7 days (commercial) - Differentiation time + 10 days (in house) | - Differentiation time + 14-30 days |
|  | Culture medium | - Manufacturer instructions | - Manufacturer instructions (commercial) - Standard culture medium (in house) | - Standard culture medium (in house) |
|  | Quality control | - Response to 100 nM digoxin; 30 nM nifedipine | - Response to 30 nM nifedipine - Cell purity >90% by a-actinin (in house) - Spontaneous beat rate ≤1.5 Hz (in house) | - Response to 100 nM isoprenaline and 30 nM nifedipine - Cell purity >70% by cTnT (for in house hiPSC-CMs) - Minimum force: 0.1 mN |
| *Experimental set-up* | Medium | - Commercial medium (serum free) | - As for TTM (commercial) - 1.8 mM Ca^2+^ (serum/protein free) (in house) | - 0.6-1 mM Ca^2+^-modified Tyrode’s soultion/DMEM (serum/protein free) |
|  | Cell loading | - ANINNE-6 plus, Rhod 3 AM and CellMask Deep Red | - FluoVolt™ | - Lentiviral mediated expression of GCaMP6f in EHTs (transduction during EHT fabrication) |
|  | Recording: Baselines | - Paced at 1.2 Hz; 7 sec/area | - Paced at 1.2-1.7 Hz; 10 sec/area | - Paced at 1.5x basal rate (0.7-2.3 Hz); 10 sec/EHT |
|  | Recording: Conc-response curves | - Replicates: 5 wells/compound and 3 areas/well + time matched vehicle controls | - Replicates: 5 wells/compound + time matched vehicle controls | - Replicates: 6 EHTs/compound + time matched vehicle controls |
|  |  | Solvent: max 0.1% DMSO; Treatment duration: 30 mins | | |
|  |  | Single concentration/well | | - Cumulative concentrations/EHT |
|  |  | - Paced at 1.2 Hz; 7 sec/area | - Paced at 1.2-1.7 Hz; 10 sec/well | - Paced at 1.5x basal rate (0.7-2.3 Hz); 10 sec/EHT |
| *Follow-up* | Analysis | - Analysis performed offline |  | - Analysis performed online and offline |
|  | Recovery | n/a | | - Rest and recreation for ≥2 days in standard medium |
|  | Additional testing | n/a | | - Baseline at 1.8 mM Ca^2+^-Tyrode/DMEM (spontaneous; 20 sec/EHT), compare to prior baseline to evaluate permanent damage |
|  | Repeat testing | No repeated use of cells | | |

**
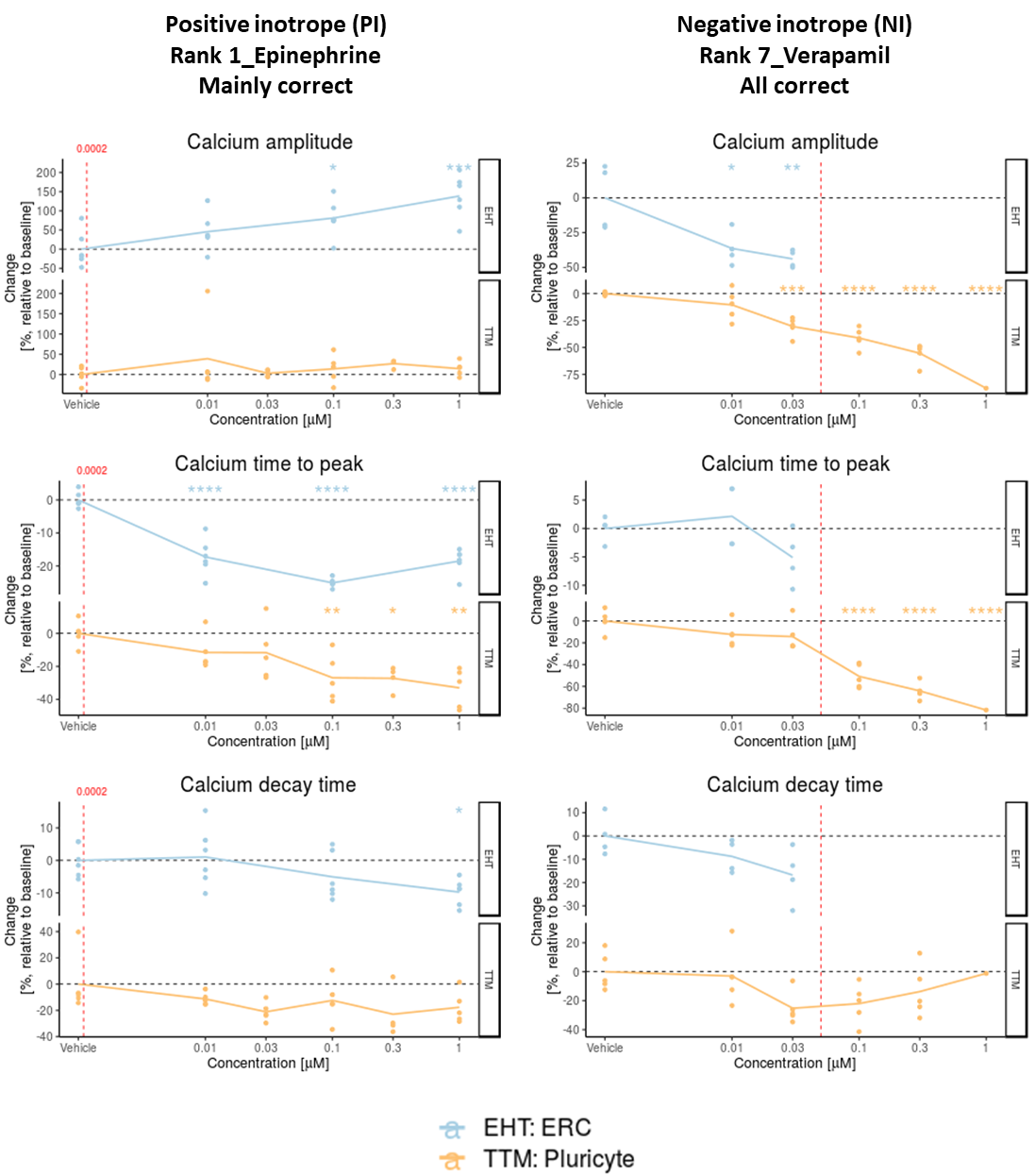
**

**Supplementary Figure 4A. Role of Ca^2+^ analysis in prediction of inotropic response.** In some instances, Ca^2+^ transient analysis was used to guide prediction of whether a compound had a positive or negative inotropic effect. Red dotted line is free therapeutic plasma concentration (FTPC). Mainly correct: correct in most platform-cell combinations (Figure 1A), All correct: correct in all platform-cell combinations (Figure 1B). Dunnett's stats vs vehicle control: *, P<0.05; **, P<0.01; ***, P<0.001; ****, P<0.0001.

**
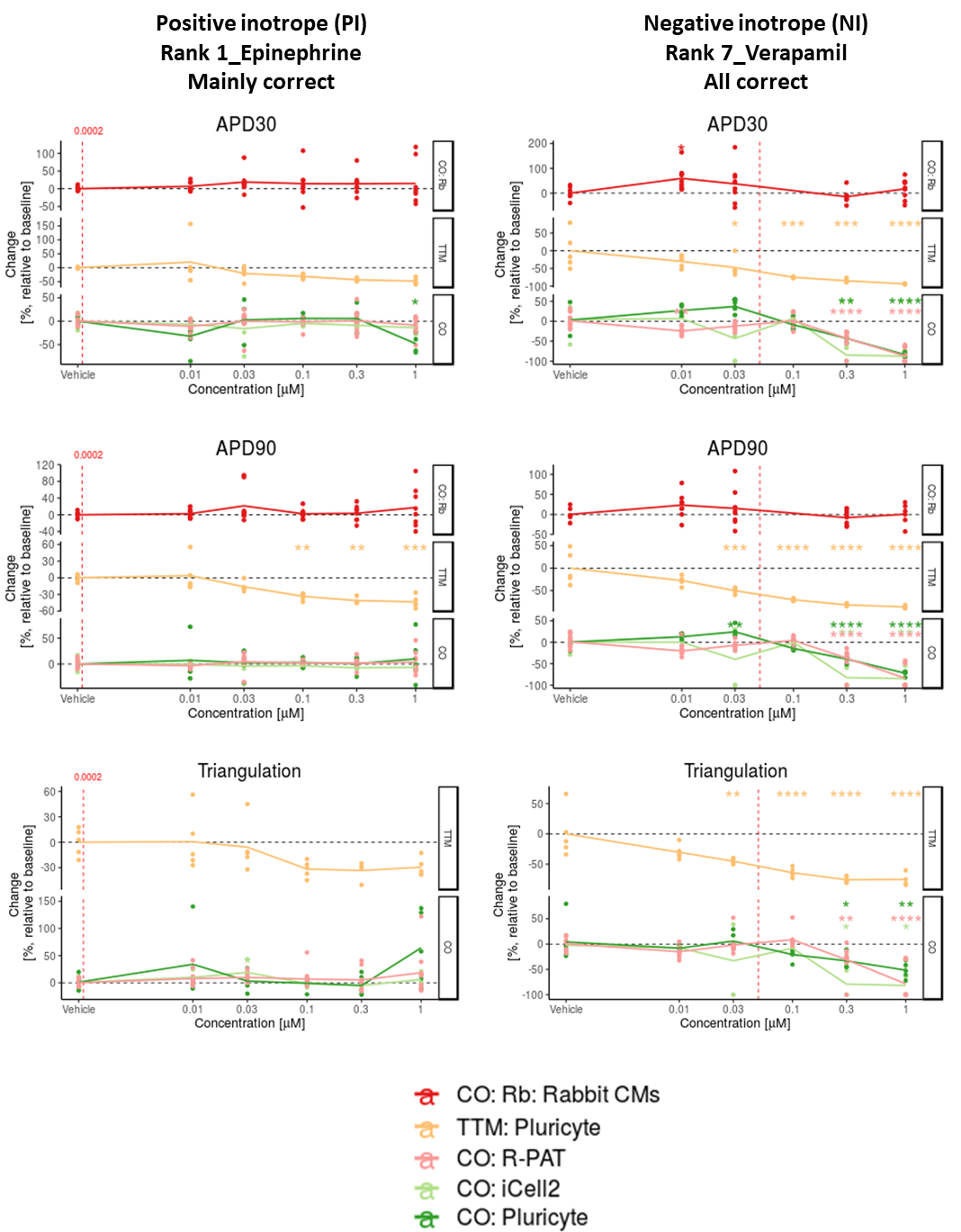
**

**Supplementary Figure 4B. Role of voltage analysis in prediction of inotropic response.** In some instances, voltage transient analysis was used to guide prediction of whether a compound had a positive or negative inotropic effect. Red dotted line is free therapeutic plasma concentration (FTPC). Dunnett's stats vs vehicle control: *, P<0.05; **, P<0.01; ***, P<0.001; ****, P<0.0001.


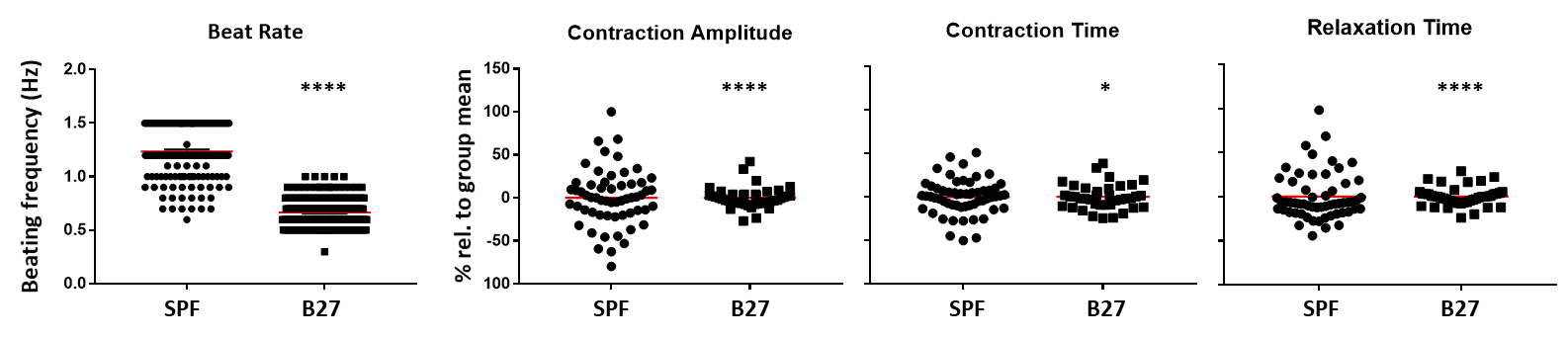


**Supplementary Figure 5. Serum/protein-free medium increases spontaneous beating rates and data distribution.** Using the CO:R-PAT platform-cell combination, recordings were evaluated in hiPSC-CMs cultured as 2D monolayers in serum-/protein-free medium (SPF) versus the protein-containing medium, RPMI-B27 (B27). Culture in SPF significantly increased spontaneous beating rates (P≤ 0.0001, Mann Whitney), and increased variance of data distribution for CA, CT and RT (P≤ 0.0001, 0.05 and 0.0001, respectively; F-test for variance)

**
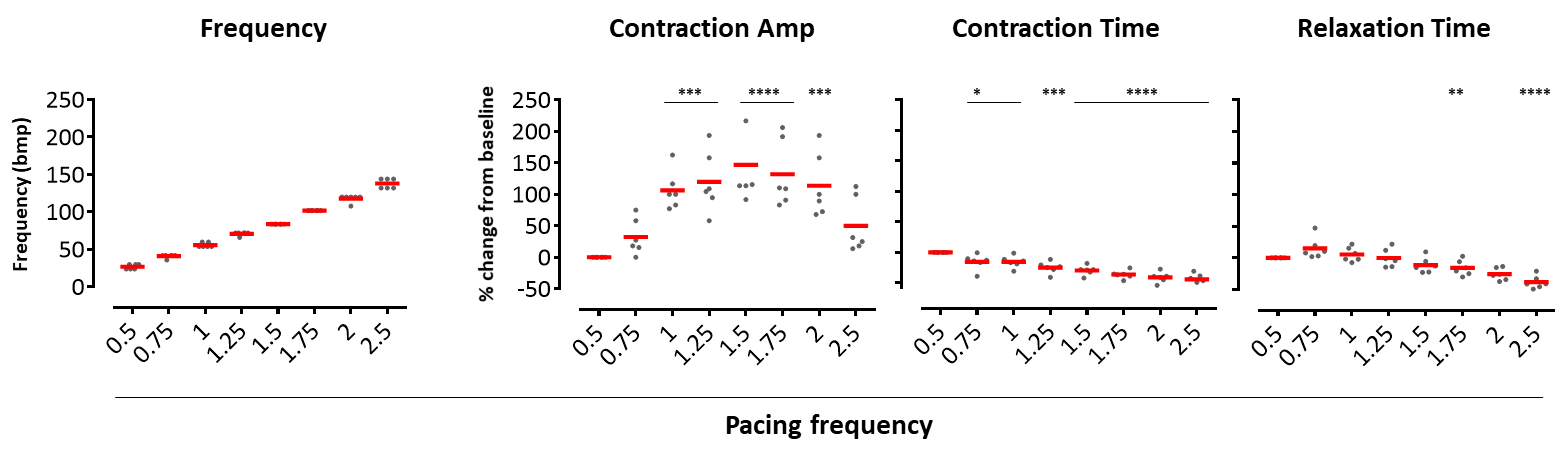
**

**Supplementary Figure 6. Positive force frequency relationship in EHTs paced between 0.5 and 1.5 Hz.** Spontaneous beat rate of EHTs was reduced by 3 hour pre-treatment with 300 nM ivabradine, an I_f_ current inhibitor. Subsequent electrical pacing showed beating frequencies that followed the frequency of the stimulus. CA increased from 0.5 to 1.5 Hz but plateaued and declined at higher frequencies. These changes were reflected in the CT and RT data. Dunnett's stats vs vehicle control: *, P<0.05; **, P<0.01; ***, P<0.001; ****, P<0.0001.

**
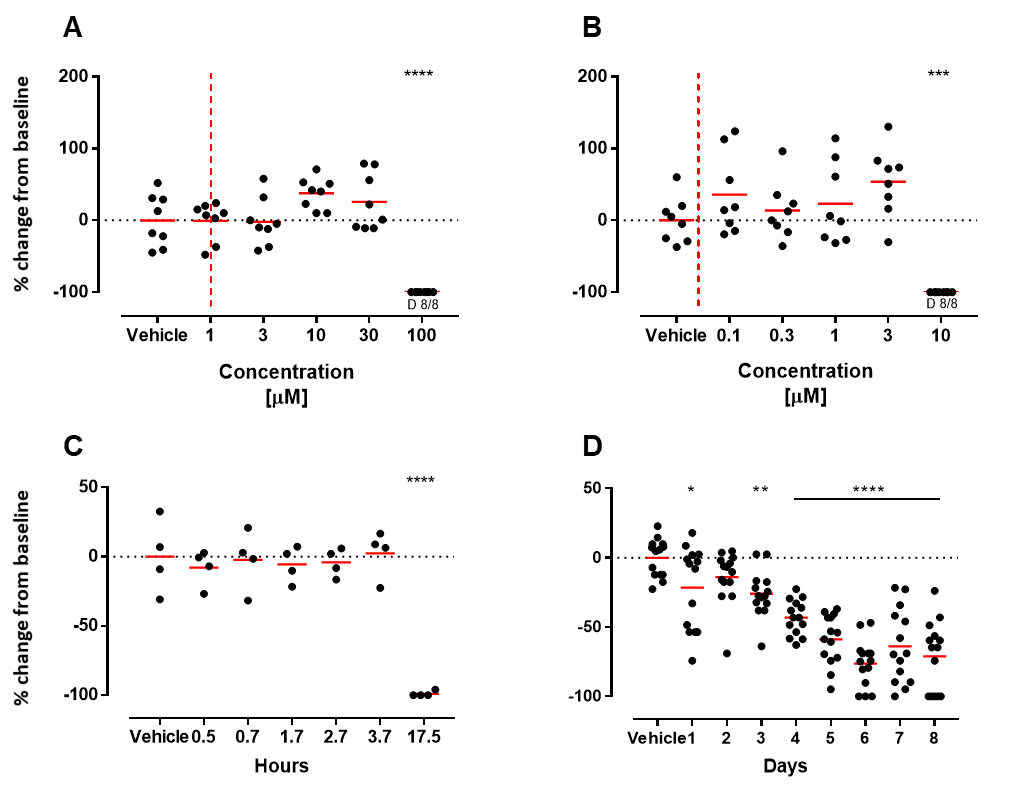
**

**Supplementary Figure 7. Prolonged exposure detects NIs in 2D and 3D cultures of hiPSC-CMs.** Contraction amplitude (CA) was measured in the CO:R-PAT platform-cell combination exposed to various concentrations of doxorubicin (A) or sunitinib (B) for 24 hours, with the highest test concentration causing cell death. Red dotted line is free therapeutic plasma concentration (FTPC). The EHT:ERC platform-cell combination was exposed to 100 µM doxorubicin (C) or 1 µM sunitinib (D) for up to 17.5 hr (C) or up to 8 days (D). CA is shown normalized to time controls. Dunnett's stats vs vehicle control: *, P<0.05; **, P<0.01; ***, P<0.001; ****, P<0.0001.

**Supplementary Table 3. Characterisation of the cell populations used relating to cardiomyocyte purity, subtype and electrophysiology**

***** Data taken from Cellular Dynamics International website ([*https://fujifilmcdi.com/products-services/icell-products/icell-cardiomyocytes2/*](https://fujifilmcdi.com/products-services/icell-products/icell-cardiomyocytes2/)) and/or PMID: 21890694

**‡** Ratio APD_90_/_50_, wherein a value of >1.7 predicts atrial-, 1.4-1.7 pacemaker- and <1.4 ventricular-like subtypes, as defined in PMID: 17895862

**§** Measured as (APD_30–40_)/(APD_70–80_) and assigned as described in PMID: 21890694

Denoted: A, predicted as atrial-like; P, predicted as pacemaker-like; V, predicted as ventricular-like.

**†** Values given for ventricular-like cells, with subtype assignment made based on morphology of patch clamp electrophysiology waveform.

**¤** Standard QC-criteria for every Pluricyte® Cardiomyocyte batch

¥ Data from PMID: 28492526; 29925535 and/or 30416051

Ф There have been conflicting views on the value of using single cell patch clamp as a predictor of cardiac subtypes. Depending on seeding density and other variables, the AP morphology of hiPSC-CMs may have any shape including those resembling chamber-specific subtypes (PMID: 25564842).

n.d. is not done (or not known / disclosed); IHC is immunohistochemistry; Replicates: N = biological, n = technical; All ± is SEM

|  | **Cell Line Characterization** | | | | | |  | **Assay Baseline Characterization** | | | | | |
| --- | --- | --- | --- | --- | --- | --- | --- | --- | --- | --- | --- | --- | --- |
| **Cell Line** | **Differentiation method** | **Purity (marker[s] used)** | **Single cell Patch Clamp** | | | |  | **Platform** | **Cell line** | **Contraction duration** | **Contraction time** | **Relaxation time** | **Beat rate** |
|  |  |  | **Subtypes**  **A:P:V%^Ф^** | **APD90** | **MDP** | **Spontaneous beat rate (Hz)** |  |  |  |  |  |  |  |
| **Pluricyte** | Small molecules, no genetic modification or selection. Day of differentiation n.d. | >70% cTnT^+^, of which >70% MLC2v^+¤^ | n.d. | 170±50; n=12 | -75±5; n=12 | 0.5±0.2 |  | **Triple Transient Measurement** | **Pluricyte** | 364±10 ms (N=3, n=50) | 208±7 ms (N=3, n=50) | 156±5 ms (N=3, n=50) | 1.2 Hz (paced) |
|  |  |  |  |  |  |  |  | **CellOPTIQ** |  | 658±6 ms (N=3, n=60) | 213±6 ms (N=3, n=60) | 445±7 ms (N=3, n=60) | 1.2 Hz (paced) |
| **iCell^2^** | n.d. | >90% a-actinin^+^; n.d. for MLC2v^+¤^ | 24:22:54%; N=59^§^ | 415±22; n=32*^†^ | -76±22; n=32^*†^ | 0.6±0.1; n=32^*†^ |  |  | **iCell^2^** | 414±3 ms (N=3, n=60) | 146±4 ms (N=3, n=60) | 267±4 ms (N=3, n=60) | 1.5-1.7 Hz (paced) |
| **R-PAT** | Small molecules and growth factors, no genetic modification or selection. Day 22-35 of differentiation | >80% a-actinin^+^ and/or cTnT^+^; >80% MLC2v^+¤^ | 4:18:78%; N=5, n=49^‡^ | 224±14; N=5, n=49 | -62±1; N=5, n=49 | n/a; triggered |  |  | **R-PAT** | 423±4 ms (N=3, n=60) | 152±2 ms (N=3, n=60) | 276±4 ms (N=3, n=60) | 1.2-1.5 Hz (paced |
|  |  |  |  |  |  |  |  | **Engineered Heart Tissue** |  | 499±11, N=144 | 183±4 ms, N=144 | 294±3 ms,  N=144 | 1.0 Hz (paced) |
| **ERC** | Small molecule & growth factor based 3D^¥^. Day 22-35 of differentiation | >80% cTnT^+^; by IHC: MLC2v, high; MLC2a, low^¥^ | n.d. | 302±20 ms, N=11^¥^ | -78±3.5 mV, N=5^¥^ | n/a; triggered 1 Hz^¥^ |  |  | **ERC** | 313±2 ms, N=134 | 166±1 ms, N=134 | 148±1 ms,  N=134 | 1.4 Hz (paced) |

Aass H, Skomedal T, Osnes JB. 1988. Increase of cyclic amp in subcellular fractions of rat heart muscle after beta-adrenergic stimulation: Prenalterol and isoprenaline caused different distribution of bound cyclic amp. J Mol Cell Cardiol. 20(9):847-860.

Abi-Gerges N, Pointon A, Pullen GF, Morton MJ, Oldman KL, Armstrong D, Valentin JP, Pollard CE. 2013. Preservation of cardiomyocytes from the adult heart. J Mol Cell Cardiol. 64:108-119.

Angus JA, Sarsero D, Fujiwara T, Molenaar P, Xi Q. 2000. Quantitative analysis of vascular to cardiac selectivity of l- and t-type voltage-operated calcium channel antagonists in human tissues. Clin Exp Pharmacol Physiol. 27(12):1019-1021.

Bailey JM, Levy JH, Kikura M, Szlam F, Hug CC, Jr. 1994. Pharmacokinetics of intravenous milrinone in patients undergoing cardiac surgery. Anesthesiology. 81(3):616-622.

Baudet S, Shaoulian R, Bers DM. 1993. Effects of thapsigargin and cyclopiazonic acid on twitch force and sarcoplasmic reticulum ca2+ content of rabbit ventricular muscle. Circ Res. 73(5):813-819.

Bellmann R. 2007. Clinical pharmacokinetics of systemically administered antimycotics. Curr Clin Pharmacol. 2(1):37-58.

Bello CL, Sherman L, Zhou J, Verkh L, Smeraglia J, Mount J, Klamerus KJ. 2006. Effect of food on the pharmacokinetics of sunitinib malate (su11248), a multi-targeted receptor tyrosine kinase inhibitor: Results from a phase i study in healthy subjects. Anticancer Drugs. 17(3):353-358.

Berger C, Meyer W, Scholz H, Starbatty J. 1985. Effects of the benzimidazole derivatives pimobendan and 2-(4-hydroxy-phenyl)-5-(5-methyl-3-oxo-4,5-dihydro-2h-6- pyridazinyl) benzimidazole . Hcl on phosphodiesterase activity and force of contraction in guinea-pig hearts. Arzneimittelforschung. 35(11):1668-1673.

Bois P, Bescond J, Renaudon B, Lenfant J. 1996. Mode of action of bradycardic agent, s 16257, on ionic currents of rabbit sinoatrial node cells. British journal of pharmacology. 118(4):1051-1057.

Boknik P, Neumann J, Kaspareit G, Schmitz W, Scholz H, Vahlensieck U, Zimmermann N. 1997. Mechanisms of the contractile effects of levosimendan in the mammalian heart. J Pharmacol Exp Ther. 280(1):277-283.

Boldt A, Gergs U, Ponicke K, Simm A, Silber RE, Neumann J. 2010. Inotropic effects of ivabradine in the mammalian heart. Pharmacology. 86(5-6):249-258.

Bristow MR, Ginsburg R, Strosberg A, Montgomery W, Minobe W. 1984. Pharmacology and inotropic potential of forskolin in the human heart. J Clin Invest. 74(1):212-223.

Brown L, Nabauer M, Erdmann E. 1986. The positive inotropic response to milrinone in isolated human and guinea pig myocardium. Naunyn-Schmiedeberg's archives of pharmacology. 334(2):196-201.

Brown L, Nabauer M, Erdmann E. 1987. Dobutamine: Positive inotropy by nonselective adrenoceptor agonism in isolated guinea pig and human myocardium. Naunyn Schmiedebergs Arch Pharmacol. 335(4):385-390.

Brown RD, Wilson JT, Kearns GL, Eichler VF, Johnson VA, Bertrand KM. 1992. Single-dose pharmacokinetics of ibuprofen and acetaminophen in febrile children. Journal of clinical pharmacology. 32(3):231-241.

Chaban R, Kornberger A, Branski N, Buschmann K, Stumpf N, Beiras-Fernandez A, Vahl CF. 2017. In-vitro examination of the positive inotropic effect of caffeine and taurine, the two most frequent active ingredients of energy drinks. BMC cardiovascular disorders. 17(1):220.

Choi HY, Bae KS, Cho SH, Ghim JL, Choe S, Jung JA, Lim HS. 2016. Population plasma and urine pharmacokinetics of ivabradine and its active metabolite s18982 in healthy korean volunteers. Journal of clinical pharmacology. 56(4):439-449.

Chu KM, Shieh SM, Hu OY. 1995. Pharmacokinetics and pharmacodynamics of enantiomers of pimobendan in patients with dilated cardiomyopathy and congestive heart failure after single and repeated oral dosing. Clin Pharmacol Ther. 57(6):610-621.

Cleland JG, Teerlink JR, Senior R, Nifontov EM, Mc Murray JJ, Lang CC, Tsyrlin VA, Greenberg BH, Mayet J, Francis DP et al. 2011. The effects of the cardiac myosin activator, omecamtiv mecarbil, on cardiac function in systolic heart failure: A double-blind, placebo-controlled, crossover, dose-ranging phase 2 trial. Lancet. 378(9792):676-683.

Costantine MM, Cleary K, Hebert MF, Ahmed MS, Brown LM, Ren Z, Easterling TR, Haas DM, Haneline LS, Caritis SN et al. 2016. Safety and pharmacokinetics of pravastatin used for the prevention of preeclampsia in high-risk pregnant women: A pilot randomized controlled trial. Am J Obstet Gynecol. 214(6):720 e721-720 e717.

Danesi R, Conte PF, Del Tacca M. 1999. Pharmacokinetic optimisation of treatment schedules for anthracyclines and paclitaxel in patients with cancer. Clinical pharmacokinetics. 37(3):195-211.

de Zeeuw S, Trines SA, Krams R, Duncker DJ, Verdouw PD. 2000. In vivo evidence that emd 57033 restores myocardial responsiveness to intracoronary ca(2+) in stunned myocardium. European journal of pharmacology. 403(1-2):99-109.

Duran JM, Makarewich CA, Trappanese D, Gross P, Husain S, Dunn J, Lal H, Sharp TE, Starosta T, Vagnozzi RJ et al. 2014. Sorafenib cardiotoxicity increases mortality after myocardial infarction. Circ Res. 114(11):1700-1712.

Dyreborg A, Krogh N, Backer V, Rzeppa S, Hemmersbach P, Hostrup M. 2016. Pharmacokinetics of oral and inhaled terbutaline after exercise in trained men. Front Pharmacol. 7:150.

Fedorov VV, Sharifov OF, Piatakova OP, Beloshapko GG, Iushmanova AV, Rozenshtraukh LV. 2002. [effects of ryanodine receptors block on spontaneous initiation of atrial fibrillation in the intact canine heart]. Kardiologiia. 42(2):59-71.

Forsberg T, Lindbom LO. 1983. Cardiovascular effects of zimelidine and tricyclic antidepressants in conscious rats. Acta Pharmacol Toxicol (Copenh). 53(3):223-229.

Giacomini KM, Massoud N, Wong FM, Giacomini JC. 1984. Decreased binding of verapamil to plasma proteins in patients with liver disease. J Cardiovasc Pharmacol. 6(5):924-928.

Giudicelli JF, Richer C, Mattei A. 1987. Pharmacokinetics and biological effects of captopril and hydrochlorothiazide after acute and chronic administration either alone or in combination in hypertensive patients. British journal of clinical pharmacology. 23 Suppl 1:51S-63S.

Hajjar RJ, DiSalvo TG, Schmidt U, Thaiyananthan G, Semigran MJ, Dec GW, Gwathmey JK. 1997. Clinical correlates of the myocardial force-frequency relationship in patients with end-stage heart failure. The Journal of heart and lung transplantation : the official publication of the International Society for Heart Transplantation. 16(11):1157-1167.

Hasenfuss G, Pieske B, Castell M, Kretschmann B, Maier LS, Just H. 1998. Influence of the novel inotropic agent levosimendan on isometric tension and calcium cycling in failing human myocardium. Circulation. 98(20):2141-2147.

Head SA, Shi W, Zhao L, Gorshkov K, Pasunooti K, Chen Y, Deng Z, Li RJ, Shim JS, Tan W et al. 2015. Antifungal drug itraconazole targets vdac1 to modulate the ampk/mtor signaling axis in endothelial cells. Proc Natl Acad Sci U S A. 112(52):E7276-7285.

Henderson KA, Borders RB, Ross JB, Huwar TB, Travis CO, Wood BJ, Ma ZXJ, Hong SP, Vinci TM, Roche BM. 2013. Effects of tyrosine kinase inhibitors on rat isolated heart function and protein biomarkers indicative of toxicity. Journal of Pharmacological and Toxicological Methods. 68(1):150-159.

Hofling B, Bolte HD. 1981. Acute negative inotropic effect of adriamycin (doxorubicin). Naunyn-Schmiedeberg's archives of pharmacology. 317(3):252-256.

Honerjager P, Heiss A, Schafer-Korting M, Schonsteiner G, Reiter M. 1984. Ud-cg 115--a cardiotonic pyridazinone which elevates cyclic amp and prolongs the action potential in guinea-pig papillary muscle. Naunyn-Schmiedeberg's archives of pharmacology. 325(3):259-269.

Horvath B, Szentandrassy N, Veress R, Almassy J, Magyar J, Banyasz T, Toth A, Papp Z, Nanasi PP. 2017. Frequency-dependent effects of omecamtiv mecarbil on cell shortening of isolated canine ventricular cardiomyocytes. Naunyn Schmiedebergs Arch Pharmacol. 390(12):1239-1246.

Ishihata A, Kushida H, Endoh M. 1988. Enantiomers of dobutamine increase the force of contraction via beta adrenoceptors, but antagonize competitively the positive inotropic effect mediated by alpha-1 adrenoceptors in the rabbit ventricular myocardium. J Pharmacol Exp Ther. 246(3):1080-1087.

Jarrott B, Louis WJ, Summers RJ. 1979. The characteristics of [3h]-clonidine binding to an alpha-adrenoceptor in membranes from guinea-pig kidney. British journal of pharmacology. 65(4):663-670.

Kamali F, Thomas SH, Ferner RE. 1993. Paracetamol elimination in patients with non-insulin dependent diabetes mellitus. British journal of clinical pharmacology. 35(1):58-61.

Kaumann AJ, Blinks JR. 1980. Stimulant and depressant effects of beta-adrenoceptor blocking agents on isolated heart muscle. A positive inotropic effect not mediated through andrenoceptors. Naunyn-Schmiedeberg's archives of pharmacology. 311(3):205-218.

Kikura M, Takada T, Sato S. 2004. Age- and sex-specific incidence, risk, and latency period of a perioperative acute thromboembolism syndrome (pats). Thromb Haemost. 91(4):725-732.

Kim A, Dombi E, Tepas K, Fox E, Martin S, Wolters P, Balis FM, Jayaprakash N, Turkbey B, Muradyan N et al. 2013. Phase i trial and pharmacokinetic study of sorafenib in children with neurofibromatosis type i and plexiform neurofibromas. Pediatric blood & cancer. 60(3):396-401.

Kirby MS, Sagara Y, Gaa S, Inesi G, Lederer WJ, Rogers TB. 1992. Thapsigargin inhibits contraction and ca2+ transient in cardiac cells by specific inhibition of the sarcoplasmic reticulum ca2+ pump. J Biol Chem. 267(18):12545-12551.

Kleiber N, Mathot RAA, Ahsman MJ, Wildschut ED, Tibboel D, de Wildt SN. 2017. Population pharmacokinetics of intravenous clonidine for sedation during paediatric extracorporeal membrane oxygenation and continuous venovenous hemofiltration. British journal of clinical pharmacology. 83(6):1227-1239.

Lasseter KC, Levey GS, Palmer RF, McCarthy JS. 1972. The effect of sulfonylurea drugs on rabbit myocardial contractility, canine purkinje fiber automaticity, and adenyl cyclase activity from rabbit and human hearts. The Journal of clinical investigation. 51(9):2429-2434.

Lemoine H, Schonell H, Kaumann AJ. 1988. Contribution of beta 1- and beta 2-adrenoceptors of human atrium and ventricle to the effects of noradrenaline and adrenaline as assessed with (-)-atenolol. British journal of pharmacology. 95(1):55-66.

Levey GS, Palmer RF, Lasseter KC, McCarthy J. 1971. Effect of tolbutamide on adenyl cyclase in rabbit and human heart and contractility of isolated rabbit atria. J Clin Endocrinol Metab. 33(2):371-374.

Lindbom LO, Forsberg T. 1981. Cardiovascular effects of zimelidine and other antidepressant in conscious rats. Acta Psychiatr Scand Suppl. 290:380-384.

Liu LC, Dorhout B, van der Meer P, Teerlink JR, Voors AA. 2016. Omecamtiv mecarbil: A new cardiac myosin activator for the treatment of heart failure. Expert Opin Investig Drugs. 25(1):117-127.

Lullmann H, Ravens U. 1973. The time courses of the changes in contractile force and in transmembfane potentials induced by cardiac glycosides in guinea-pig papillary muscle. Br J Pharmacol. 49(3):377-390.

Mahoney L, Shah G, Crook D, Rojas-Anaya H, Rabe H. 2016. A literature review of the pharmacokinetics and pharmacodynamics of dobutamine in neonates. Pediatr Cardiol. 37(1):14-23.

Mannhardt I, Eder A, Dumotier B, Prondzynski M, Kramer E, Traebert M, Sohren KD, Flenner F, Stathopoulou K, Lemoine MD et al. 2017. Blinded contractility analysis in hipsc-cardiomyocytes in engineered heart tissue format: Comparison with human atrial trabeculae. Toxicological sciences : an official journal of the Society of Toxicology. 158(1):164-175.

Marks L, Borland S, Philp K, Ewart L, Lainee P, Skinner M, Kirk S, Valentin JP. 2012. The role of the anaesthetised guinea-pig in the preclinical cardiac safety evaluation of drug candidate compounds. Toxicol Appl Pharmacol. 263(2):171-183.

Matsushita T, Okamato M, Toyama J, Kodama I, Ito S, Fukutomi T, Suzuki S, Itoh M. 2000. Adriamycin causes dual inotropic effects through complex modulation of myocardial ca2+ handling. Jpn Circ J. 64(1):65-71.

McQuinn RL, Pentikainen PJ, Chang SF, Conard GJ. 1988. Pharmacokinetics of flecainide in patients with cirrhosis of the liver. Clinical pharmacology and therapeutics. 44(5):566-572.

Mebazaa A, Nieminen MS, Packer M, Cohen-Solal A, Kleber FX, Pocock SJ, Thakkar R, Padley RJ, Poder P, Kivikko M et al. 2007. Levosimendan vs dobutamine for patients with acute decompensated heart failure: The survive randomized trial. JAMA : the journal of the American Medical Association. 297(17):1883-1891.

Mesirca P, Alig J, Torrente AG, Muller JC, Marger L, Rollin A, Marquilly C, Vincent A, Dubel S, Bidaud I et al. 2014. Cardiac arrhythmia induced by genetic silencing of 'funny' (f) channels is rescued by girk4 inactivation. Nat Commun. 5:4664.

Molenaar P, Savarimuthu SM, Sarsero D, Chen L, Semmler AB, Carle A, Yang I, Bartel S, Vetter D, Beyerdorfer I et al. 2007. (-)-adrenaline elicits positive inotropic, lusitropic, and biochemical effects through beta2 -adrenoceptors in human atrial myocardium from nonfailing and failing hearts, consistent with gs coupling but not with gi coupling. Naunyn Schmiedebergs Arch Pharmacol. 375(1):11-28.

Mooney L, Skinner M, Coker SJ, Currie S. 2015. Effects of acute and chronic sunitinib treatment on cardiac function and calcium/calmodulin-dependent protein kinase ii. British journal of pharmacology. 172(17):4342-4354.

Murakami M, Furukawa Y, Karasawa Y, Ren LM, Takayama S, Chiba S. 1992. Inhibition by glibenclamide of negative chronotropic and inotropic responses to pinacidil, acetylcholine, and adenosine in the isolated dog heart. Journal of cardiovascular pharmacology. 19(4):618-624.

Naranjo CA, Sellers EM, Kaplan HL, Hamilton C, Khouw V. 1984. Acute kinetic and dynamic interactions of zimelidine with ethanol. Clinical pharmacology and therapeutics. 36(5):654-660.

Orstavik O, Ata SH, Riise J, Dahl CP, Andersen GO, Levy FO, Skomedal T, Osnes JB, Qvigstad E. 2014. Inhibition of phosphodiesterase-3 by levosimendan is sufficient to account for its inotropic effect in failing human heart. Br J Pharmacol. 171(23):5169-5181.

Papp Z, Edes I, Fruhwald S, De Hert SG, Salmenpera M, Leppikangas H, Mebazaa A, Landoni G, Grossini E, Caimmi P et al. 2012. Levosimendan: Molecular mechanisms and clinical implications: Consensus of experts on the mechanisms of action of levosimendan. Int J Cardiol. 159(2):82-87.

Perez O, Gay P, Franqueza L, Carron R, Valenzuela C, Delpon E, Tamargo J. 1995. Effects of the two enantiomers, s-16257-2 and s-16260-2, of a new bradycardic agent on guinea-pig isolated cardiac preparations. British journal of pharmacology. 115(5):787-794.

Planelles-Herrero VJ, Hartman JJ, Robert-Paganin J, Malik FI, Houdusse A. 2017. Mechanistic and structural basis for activation of cardiac myosin force production by omecamtiv mecarbil. Nat Commun. 8(1):190.

Pogatsa G, Dubecz E. 1977. The direct effect of hypoglycaemic sulphonylureas on myocardial contractile force and arterial blood pressure. Diabetologia. 13(5):515-519.

Puttonen J, Kantele S, Ruck A, Ramela M, Hakkinen S, Kivikko M, Pentikainen PJ. 2008. Pharmacokinetics of intravenous levosimendan and its metabolites in subjects with hepatic impairment. Journal of Clinical Pharmacology. 48(4):445-454.

Qu Y, Fang M, Gao B, Amouzadeh HR, Li N, Narayanan P, Acton P, Lawrence J, Vargas HM. 2013. Itraconazole decreases left ventricular contractility in isolated rabbit heart: Mechanism of action. Toxicol Appl Pharmacol. 268(2):113-122.

Rao N. 2007. The clinical pharmacokinetics of escitalopram. Clinical pharmacokinetics. 46(4):281-290.

Reyes G, Schwartz PH, Newth CJ, Eldadah MK. 1993. The pharmacokinetics of isoproterenol in critically ill pediatric patients. Journal of clinical pharmacology. 33(1):29-34.

Rosen MR, Gelband H, Hoffman BF. 1971. Effects of phentolamine on electrophysiologic properties of isolated canine purkinje fibers. The Journal of pharmacology and experimental therapeutics. 179(3):586-593.

Rydberg T, Jonsson A, Karlsson MO, Melander A. 1997. Concentration-effect relations of glibenclamide and its active metabolites in man: Modelling of pharmacokinetics and pharmacodynamics. British journal of clinical pharmacology. 43(4):373-381.

Sada H. 1978. Effect of phentolamine, alprenolol and prenylamine on maximum rate of rise of action potential in guinea-pig papillary muscles. Naunyn-Schmiedeberg's archives of pharmacology. 304(3):191-201.

Satoh E, Yanagisawa T, Taira N. 1990. Specific antagonism by glibenclamide of negative inotropic effects of potassium channel openers in canine atrial muscle. Jpn J Pharmacol. 54(2):133-141.

Schafers RF, Adler S, Daul A, Zeitler G, Vogelsang M, Zerkowski HR, Brodde OE. 1994. Positive inotropic effects of the beta 2-adrenoceptor agonist terbutaline in the human heart: Effects of long-term beta 1-adrenoceptor antagonist treatment. J Am Coll Cardiol. 23(5):1224-1233.

Stage C, Jurgens G, Guski LS, Thomsen R, Bjerre D, Ferrero-Miliani L, Lyauk YK, Rasmussen HB, Dalhoff K, Consortium I. 2017. The pharmacokinetics of enalapril in relation to ces1 genotype in healthy danish volunteers. Basic Clin Pharmacol Toxicol. 121(6):487-492.

Sugiyama A, Satoh Y, Shiina H, Takahara A, Yoneyama M, Hashimoto K. 2001. Cardiac electrophysiologic and hemodynamic effects of sildenafil, a pde5 inhibitor, in anesthetized dogs. Journal of cardiovascular pharmacology. 38(6):940-946.

Sutko JL, Willerson JT. 1980. Ryanodine alteration of the contractile state of rat ventricular myocardium. Comparison with dog, cat, and rabbit ventricular tissues. Circ Res. 46(3):332-343.

Teerlink JR, Clarke CP, Saikali KG, Lee JH, Chen MM, Escandon RD, Elliott L, Bee R, Habibzadeh MR, Goldman JH et al. 2011. Dose-dependent augmentation of cardiac systolic function with the selective cardiac myosin activator, omecamtiv mecarbil: A first-in-man study. Lancet. 378(9792):667-675.

Temma K, Akera T, Chugun A, Kondo H, Hagane K, Hirano S. 1993. Comparison of cardiac actions of doxorubicin, pirarubicin and aclarubicin in isolated guinea-pig heart. European journal of pharmacology. 234(2-3):173-181.

Walker DK, Ackland MJ, James GC, Muirhead GJ, Rance DJ, Wastall P, Wright PA. 1999. Pharmacokinetics and metabolism of sildenafil in mouse, rat, rabbit, dog and man. Xenobiotica. 29(3):297-310.

Whiting B, Williams RL, Lorenzi M, Varady JC, Robins DS. 1981. Effect of naproxen on glucose metabolism and tolbutamide kinetics and dynamics in maturity onset diabetics. British journal of clinical pharmacology. 11(3):295-302.

Witchel HJ, Pabbathi VK, Hofmann G, Paul AA, Hancox JC. 2002. Inhibitory actions of the selective serotonin re-uptake inhibitor citalopram on herg and ventricular l-type calcium currents. FEBS letters. 512(1-3):59-66.

Wortsman J, Frank S, Cryer PE. 1984. Adrenomedullary response to maximal stress in humans. Am J Med. 77(5):779-784.
